# Comparative HIV-1 Phylogenies Characterized by *PR/RT, Pol* and Near-Full-Length Genome Sequences

**DOI:** 10.3390/v14102286

**Published:** 2022-10-17

**Authors:** Cicek Topcu, Vasilis Georgiou, Johana Hezka Rodosthenous, Leondios G. Kostrikis

**Affiliations:** 1 Department of Biological Sciences, University of Cyprus, 1 University Avenue, Aglantzia, 2109 Nicosia, Cyprus; 2 Cyprus Academy of Sciences, Letters, and Arts, 60–68 Phaneromenis Street, 1011 Nicosia, Cyprus

**Keywords:** human immunodeficiency virus type 1, HIV-1 genotypic subtyping, near-full-length HIV-1 genome sequencing, HIV-1 genetic diversity, HIV-1 phylogeny, Cyprus

## Abstract

In an effort to evaluate the accuracy of HIV-1 phylogenies based on genomes of increasing length, we developed a comprehensive near-full-length HIV-1 genome RT–PCR assay and performed a comparative evaluation via phylogenetic analyses. To this end, we conducted comparative analyses of HIV-1 phylogenies derived based on HIV-1 *PR/RT* (2253–3359 in the HXB2 genome) and *pol* region (2253–5250 in the HXB2 genome) sequences isolated from 134 HIV-1-infected patients in Cyprus (2017–2019). The HIV-1 genotypic subtypes determined using six subtyping tools (REGA 3.0, COMET 2.3, jpHMM, SCUEAL, Stanford, and Geno2pheno) were compared to investigate the discrepancies generated among different tools. To evaluate the accuracy of defined HIV-1 phylogenies, the samples exhibiting at least one discrepant subtyping result among different subtyping tools in both *PR/RT* and *pol* regions or only in the *pol* region (*n* = 38) were selected for near-full-length HIV-1 genome (790–8795 in HXB2 genome) sequencing using a newly developed RT–PCR/sequencing assay. The obtained sequences were employed for HIV-1 genotypic subtype determination and subjected to comparative phylogenetic-based analyses. It was observed that 39.6% of the 134 samples presented discrepancies in the *PR/RT* region, while 28.4% presented discrepancies in the *pol* region. REGA 3.0 produced the fewest discrepancies collectively in both regions and was selected for subsequent subtyping and comparative phylogenetic analyses of near-full-length HIV-1 genome sequences. The analyses of near-full-length HIV-1 genome sequences identified 68.4% of the 38 ‘discrepant samples’ (*n* = 26) as belonging to uncharacterized recombinant HIV-1 strains, while 21.1% were circulating recombinant forms (CRFs) (*n* = 8) and 10.5% belonged to pure group M subtypes (*n* = 4). The findings demonstrated a significant reduction of 11.2% in discrepancies when *pol* region sequences were used compared to *PR/RT* region sequences, indicating that increased nucleotide sequence lengths are directly correlated with more consistent subtype classification. The results also revealed that if the discrepancy in *pol* region subtyping results persists, then there is a high likelihood (89.5%) that the query sequence is a recombinant HIV-1 strain, 68.4% of which belong to uncharacterized recombinant HIV-1 strains. The results of this study showed that REGA 3.0 presented the best performance in subtyping recombinant HIV-1 strains, while Stanford performed better in defining phylogenies of pure group M subtypes. The study highlights that, especially in populations with polyphyletic HIV-1 epidemics resulting in a high prevalence of recombinant HIV-1 strains, neither *PR/RT* nor *pol* region sequences are reliable for the determination of HIV-1 genotypic subtypes in samples showing discrepancies among different subtyping tools, and only near-full-length or full-length HIV-1 genome sequences are sufficiently accurate.

## 1. Introduction

The human immunodeficiency virus type 1 (HIV-1) epidemic has been a global health threat for over four decades, with 37.7 million people currently living with such infections [1]. Since the development of highly active antiretroviral therapy (HAART), HIV viremia has almost been brought to an undetectable level achieving a significant decrease in acquired immunodeficiency syndrome (AIDS)-related mortality and a longer life span, although some degree of morbidity still exists in HIV-infected patients receiving therapy due to incomplete recovery of the immune system [2,3]. One of the key scientific challenges for the effective treatment and prevention of HIV-1 infection is the high degree of genetic variability of the virus acquired via fast viral turnover and a high mutation frequency [4]. Due to its broad genetic diversity, HIV-1 is classified into four groups (M, N, O, and P), which are further subdivided into 10 distinct phylogenetic subtypes (A, B, C, D, F, G, H, J, K, and L) within the major group, M [5,6].

As a consequence of the geographic location of Cyprus at the crossroads of three continents, Africa, Europe, and Asia, and the consequent import of numerous HIV-1 group M subtypes and circulating recombinant forms (CRFs) from these continents, the HIV-1 epidemic on the island has become highly polyphyletic [7]. The most recently published study from Cyprus (2010–2012) showed that the main circulating HIV-1 strains were subtype B (41.0%), A1 (19.0%), C (7.0%), F1 (8.0%), CRF02_AG (4%), A2 (2.0%), other CRFs (7.0%) and uncharacterized HIV-1 recombinant forms (12%) [8]. Although the preliminary results of a prospective molecular epidemiology study (2017–2021) (C. Topcu et al., manuscript in preparation for publication) indicate that polyphyletic infections prevail in Cyprus, sub-subtype A1 has become the predominant subtype (41%), followed by subtype B (33%). Therefore, the possible coinfection of an individual with two or more divergent HIV-1 strains in a polyphyletic infection, accompanied by the high recombinogenic nature of HIV-1, can lead to the emergence of new recombinant HIV-1 strains [9]. The continuous emergence and transmission of HIV-1 recombinants have led to the identification and characterization of 132 well-established CRFs, as reported in the Los Alamos HIV Sequence Database [10], and many other uncharacterized recombinant HIV-1 strains. In fact, the investigation of HIV-1 *pol* region sequences in Cyprus revealed a 10% increase in the prevalence of uncharacterized HIV-1 recombinants, from 12% to approximately 22% (C. Topcu et al., manuscript in preparation for publication).

Currently, there is a significant effort by the scientific community to perform genotypic analyses based on sequences that are commonly derived from drug resistance analyses. The most abundant sequences recorded since the standardization of drug resistance analyses in the early 2000s have been *protease/reverse transcriptase* (*PR/RT)* region sequences [11]. More recently, *pol* region sequences that include *integrase (IN)* and *PR/RT* have become widely available with the introduction of integrase inhibitors in first-line regimens in 2019 [12]. Therefore, many laboratories around the world are utilizing these sequences to derive HIV-1 phylogenies as a secondary effort. Nevertheless, especially in HIV-1 epidemics with broad genetic diversity and a high percentage of uncharacterized HIV-1 recombinants, the accuracy and reliability of the subtyping results based on partial sequences are uncertain. Due to the mosaic genomic structure of recombinant HIV-1 strains, HIV-1 genotypic subtypes differ based on the length of the nucleotide sequence, the region of the HIV-1 genome being analyzed, and the HIV-1 genotypic subtyping tool used.

As such, we have developed a comprehensive near-full-length HIV-1 genome RT–PCR assay in an effort to delineate the accuracy of phylogenies derived based on HIV-1 *PR/RT* and *pol* region (*PR, RT, IN* and partial *vif*) sequences and performed a comparative evaluation using state-of-the-art phylogenetic analysis methods. In this study, we compared HIV-1 phylogenies derived from six different HIV-1 subtyping tools based on the two regions using a cohort presenting high genetic diversity [13]. Subsequently, we demonstrated the correlation between the consistency of subtype classification among different tools and the length of nucleotide sequences being analyzed by investigating the number of discrepancies generated among the subtyping tools. Moreover, we evaluated the accuracy of the subtyping results of samples that presented discrepancies among different tools, determined based on partial HIV-1 sequences in comparison to near-full-length HIV-1 genome sequences. The findings of this study highlight a significant reduction in the discrepancies generated among different subtyping tools when *pol* region sequences are used compared to *PR/RT* region sequences. Despite the decreased error rate, if the discrepancy in the subtyping results of *pol* region sequences persists, there is a high likelihood that the query sequence belongs to a recombinant HIV-1 strain. The subtyping results and the comparative phylogenetic analyses of near-full-length HIV-1 genome sequences showed that neither *PR/RT* nor *pol* region sequences are representative of the HIV-1 genotypic subtypes of the entire genome, especially in recombinant HIV-1 strains, but only near-full-length or full-length HIV-1 genome sequences are sufficiently accurate. Among the tested HIV-1 subtyping tools, REGA 3.0 showed better performance in the subtype determination of recombinant HIV-1 strains, while Stanford was found to more correctly define HIV-1 phylogenies belonging to pure group M subtypes. Furthermore, this study highlights the significance of near-full-length or full-length HIV-1 genome sequencing to understand the extent of genetic diversity, particularly in polyphyletic HIV-1 epidemics, in addition to evaluating the actual prevalence of uncharacterized HIV-1 recombinants for the characterization of potential novel unique recombinant forms (URFs) and CRFs.

## 2. Materials and Methods

### 2.1. Study Participants and Sample Requirements

In an effort to elucidate the phylogenies derived from *PR/RT* and *pol* regions in the present study, we included HIV-1 nucleotide sequences isolated from 134 newly diagnosed or chronically HIV-1-infected patients from 9 March 2017 to 1 August 2019 in Cyprus. All experimental procedures conducted as part of this study were approved by the Cyprus National Bioethics Committee (CNBC) (approval number EEBK EΠ 23 January 2017, approval date 20 February 2017). We collected data from previously published research conducted by our laboratory according to the relevant guidelines and regulations of CNBC and the Office of the Commissioner for Personal Data Protection in Cyprus [13]. Among 144 HIV-1-infected study participants included in a previously published research project focused on the development of an HIV-1 genotypic drug resistance assay for the entire *pol* region, 134 were included in this study. The remaining 10 patient samples were excluded from this study because they were either PCR negative or failed the sequencing of the HIV-1 *env* region (5041–8795 in the HXB2 genome) because of low-quality reads. Written informed consent was obtained from all participating HIV-1-infected patients, as was a questionnaire containing detailed clinical, epidemiological, and behavioral information. The inclusion criteria of this previously published research study from which the data were collected were set based on the previously defined enrollment strategy [7]. Specifically, the inclusion criteria stated that all study participants were consenting, newly diagnosed, or chronically HIV-1-infected patients and had an HIV-1 viral load equal to or greater than 10^3^ RNA copies/mL plasma [13]. Moreover, chronically HIV-1-infected patients were included in this study irrespective of the patients’ therapy status indicating whether the patient had ever received combination antiretroviral therapy (cART). Additionally, 124 newly diagnosed or chronic antiretroviral-naïve HIV-1-infected patients among the 134 participants included in this study were also included in a prospective molecular epidemiology study (C. Topcu et al., manuscript in preparation for publication) conducted by our laboratory. The prospective molecular epidemiology study (C. Topcu et al., manuscript in preparation for publication), examining molecular epidemiology, HIV-1 transmission dynamics, and transmitted drug resistance in Cyprus, was conducted from 9 March 2017 to 14 October 2021.

Double coding was utilized to safeguard the confidentiality of the study participants. Accordingly, a unique hospital identification number and, subsequently, a unique laboratory identification number was allocated to each study participant and corresponding blood sample, respectively. The unique laboratory identification number cannot be traced back to the hospital identification number, which cannot be traced back to the identity of the patient, ensuring anonymity through double coding. The blood samples, consent forms, and questionnaires belonging to the study participants were collected at the Grigorios HIV Clinic of Larnaca General Hospital in accordance with the guidelines and regulations of CNBC. The Grigorios HIV Clinic of Larnaca General Hospital is the single clinical national service for the care of HIV-1-infected individuals in Cyprus and therefore offers a unique opportunity to provide a cohort of HIV-1 patients representative of the epidemic in Cyprus. Upon sample collection, the blood samples were sent to the Laboratory of Biotechnology and Molecular Virology of the University of Cyprus for processing.

### 2.2. Plasma Isolation and HIV-1 RNA Extraction

Blood samples (8 mL) were collected in Becton Dickinson Vacutainer^®^ Cell Preparation Tubes (CPT) at the Grigorios HIV Clinic of Larnaca General Hospital. The CPTs containing the blood samples were later transferred to the Laboratory of Biotechnology and Molecular Virology of the University of Cyprus, where they were processed within two hours of sampling. The isolation of plasma from whole blood was performed according to the previously described methodology [13]. Subsequent HIV-1 RNA extraction from the isolated plasma was conducted using the QIAamp Viral RNA Mini Kit (QIAGEN, Hilden, Germany, 52904) according to the manufacturer’s instructions. Specifically, HIV-1 RNA was extracted from 140 μL plasma, resulting in a 60 μL eluate of purified HIV-1 RNA.

### 2.3. PCR Amplification and Sanger Sequencing of the HIV-1 Pol Region

**Figure 1:**
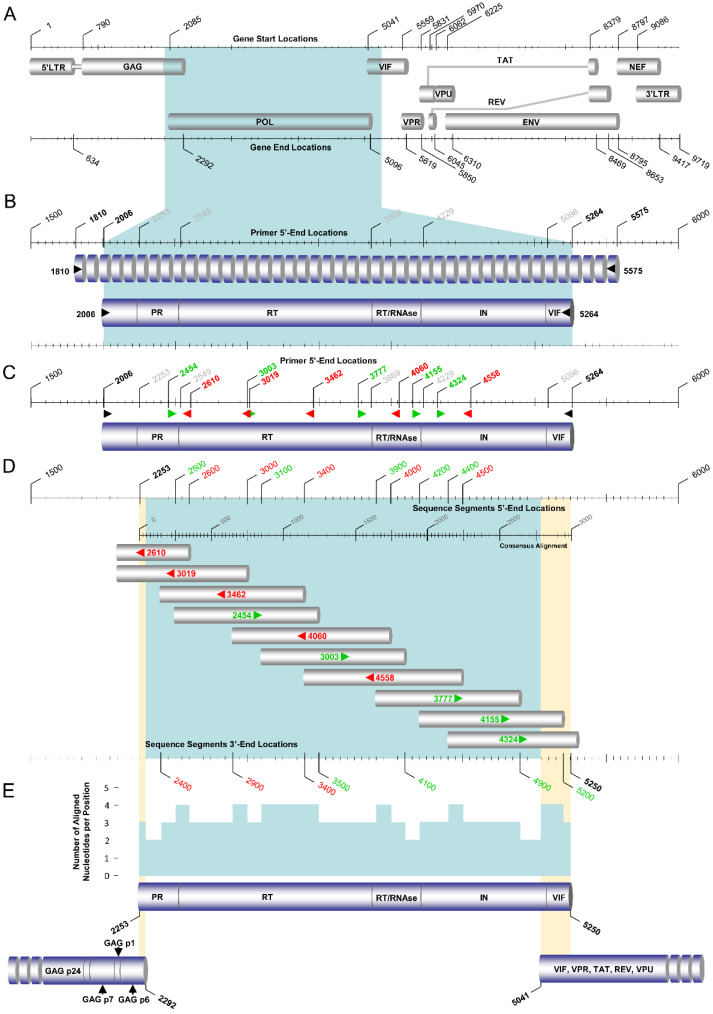
The PCR and sequencing design of the pol RT–PCR assay for the amplification and sequencing of the *pol* (2253 to 5250 in the HXB2 genome) region of HIV-1 group M subtypes, circulating recombinant forms (CRFs), and recombinants. (**A**) The top part of the schematic depicts the overall genetic organization of HIV-1 with respect to the HXB2 genome (GenBank accession number K03455), where each major genetic region is represented by labeled gray cylindrical figures. The numbers above and below the diagram show the corresponding beginning and end of these genetic regions indicated by the HXB2 genome. The shaded section, in bright blue in the HIV-1 gene map, denotes the region of amplification in this pol RT–PCR assay of HIV-1. (**B**) The second part of the schematic presents the primary RT–PCR and the secondary nested PCR design for the region of amplification. The numbers above the diagram in gray correspond to the beginning and end of the genes within the region of amplification (*PR, RT, RNAse, IN*) with respect to the HXB2 genome. The intermittent and solid cylindrical figures represent the primary RT–PCR and secondary nested PCR products, respectively. The forward and reverse primary (1810 and 5575) and secondary (2006 and 5264) PCR primers are indicated on the sides of the cylindrical figures. The orientation of the PCR primers is indicated with black arrows. The 5′-end-primer-binding positions of these PCR primers are indicated with the corresponding numbers above the diagram in bold black font, in accordance with HXB2 numbering. (**C**) The solid cylindrical figure indicates the final amplicon resulting from the secondary nested PCR, consisting of the entire *pol* and 5′ end of the *vif* regions. The 5′-end-primer-binding positions of the forward and reverse sequencing primers are indicated with the corresponding numbers above the diagram in green and red, respectively, in accordance with HXB2 numbering, while the bold black numbers indicate the secondary nested PCR primers. The orientation of the sequencing primers is also shown with the labeled green and red arrows. The numbers above the diagram in gray correspond to the beginning and end of the genes within the region of amplification (*PR, RT, RNAse, IN*) with respect to the HXB2 genome. (**D**) The gray cylindrical figures represent the sequencing primer amplicons acquired from labeled sequencing primers, and the orientation of these sequencing primers is indicated with the corresponding arrows. The numbers above and below the diagram indicate the corresponding beginning and end of the sequencing primer amplicons in accordance with HXB2 numbering. (**E**) The top solid cylindrical figure indicates the resulting aligned DNA sequence composed of all the partial sequencing primer amplicons (corresponding to nucleotides 2253 to 5250 in the HXB2 genome). This final aligned DNA sequence contains the entire *pol* region. The graph above the resulting aligned DNA sequence demonstrates the number of aligned nucleotides per position when all the partial sequencing primer amplicons are combined. The bottom left cylindrical figure indicates the 3′ end of the resulting aligned DNA sequence from the gag RT–PCR assay, while the bottom right cylindrical figure indicates the 5′ end of the resulting aligned DNA sequence from the env RT–PCR assay. The shaded sections in bright yellow represent the overlapping regions between the final amplicons of the gag and pol RT–PCR assays and the pol and env RT–PCR assays.

Initially, the *pol* region (2253–5250 in the HXB2 genome) sequences of 134 HIV-1 viral genomes isolated from 134 HIV-1-infected patients were successfully amplified according to a previously described touchdown HIV-1 pol RT–PCR assay (Figure 1) [13]. Specifically, touchdown PCR was adopted for this HIV-1 pol RT–PCR assay, which was developed as a comprehensive HIV-1 genotypic drug resistance assay for all commercially available reverse transcriptase, protease, and integrase inhibitors for maximal specificity. The pol RT–PCR assay reliably amplifies and sequences the entire *pol* region independent of HIV-1 subtypes, CRFs, and recombinants, using primers specifically designed to cover as many HIV-1 strains as possible. The assay achieves this by the addition of wobbles to any loci that are characterized by more than one nucleotide in the reference dataset of HIV-1 strains. As such, with the use of this assay, we were able to obtain a cohort representative of the polyphyletic HIV-1 epidemic of Cyprus with a diverse set of HIV-1 group M subtypes, CRFs, and other recombinants.

The PCR kits, PCR and sequencing primers, and PCR thermal cycling conditions used for the pol RT–PCR assay were previously described in detail [13]. Briefly, for the primary RT–PCR and the secondary nested PCR steps of this assay, the SuperScript™ IV One-Step RT–PCR System (ThermoFisher Scientific, Waltham, MA, USA, 12594025) and Platinum™ Hot Start PCR 2× Master Mix (ThermoFisher Scientific, Waltham, MA, USA, 13000012) were used, respectively. The primers used for the primary RT–PCR step were 1810 and 5575, and those for the secondary nested PCR step were 2006 and 5264 (Figure 1B). The PCR thermal cycling conditions of the pol RT–PCR assay are summarized in Table 1. To confirm the success of PCR, a positive RNA control was utilized, while a negative control was utilized to exclude the possibility of contamination. The secondary nested PCR step of the pol RT–PCR assay yielded a 3259 bp final product, which was then sent to Macrogen Europe (https://dna.macrogen-europe.com/eng/, accessed on 12 September 2022) for purification and Sanger sequencing. The sequencing design of the HIV-1 pol RT–PCR assay involved 10 sequencing primers: 2454, 2610, 3003, 3019, 3462, 3777, 4060, 4324, 4155, and 4558 (Figure 1C,D). The 10 sequencing primers provided approximately three-primer coverage, ranging from two to four primers at each nucleotide position to achieve reliable sequencing (Figure 1E). The sequencing primer amplicons were validated by checking the quality of the sequencing readout on Geneious^®^ 11.1.4 (https://www.geneious.com, accessed on 12 September 2022) software to obtain the consensus sequence according to a previously published protocol [13].

### 2.4. Subtyping of HIV-1 PR/RT and Pol Region Nucleotide Sequences

Thereafter, the 134 HIV-1 *pol* region nucleotide sequences obtained through Sanger sequencing were analyzed for HIV-1 genotypic subtypes based on the *PR/RT* (2253–3359 in the HXB2 genome) and *pol* (2253–5250 in the HXB2 genome) regions separately. Initially, Molecular Evolutionary Genetics Analysis (MEGA X) software was utilized to generate a multiple sequence alignment of the *pol* region nucleotide sequences using the ClustalW algorithm [14]. Multiple sequence alignment was then employed to manually extract the *PR/RT* region nucleotide sequences using AliView software [15]. The HIV-1 genotypic subtypes were determined using six different subtyping tools: REGA HIV-1 subtyping tool, version 3.0 (REGA 3.0) [16], COntext-based Modeling for Expeditious Typing version 2.3 (COMET 2.3) [17], jumping profile Hidden Markov Model (jpHMM) [18], SCUEAL [19], the HIVdb Program of the Stanford University HIV Drug Resistance Database (Stanford) [20,21], and Geno2pheno 3.4 [22].

Upon determining the HIV-1 genotypic subtypes of the 134 sequences based on the *PR/RT* (2253–3359 in the HXB2 genome) and *pol* (2253–5250 in the HXB2 genome) regions, the subtypes were analyzed for any disagreements among different subtyping tools. Any disagreements in the subtyping results generated by the aforementioned subtyping tools were considered discrepancies. The samples that showed at least one disagreement in the subtyping of the *PR/RT* region among different subtyping tools were considered to be ‘discrepant in the *PR/RT* region’. The samples that showed at least one disagreement in the subtyping of the *pol* region among different subtyping tools were considered to be ‘discrepant in the *pol* region’. The final designation of ‘discrepant sample’ was given to samples that showed at least one disagreement in both the *PR/RT* and *pol* regions or only in the *pol* region. In the following scenarios, the subtype was not included as a discrepancy: when SCUEAL generated the outcome ‘Complex’, when COMET 2.3 generated the outcome ‘unassigned’ but suggested the correct subtyping, when the constituent subtypes of a recombinant were correctly specified by any subtyping tool, and when the term ‘CRF-like’ was output instead of ‘CRF’. Eventually, the samples identified as ‘discrepant samples’ were selected for near-full-length HIV-1 genome sequencing. It is important to note that 38 samples were established as ‘discrepant samples’, whose near-full-length HIV-1 genomes were amplified and sequenced.

Furthermore, the respective total number of ‘discrepant samples’ based on the *PR/RT* and *pol* regions was calculated to show the correlation between the increasing length of nucleotide sequence and the consistency in subtypes among different subtyping tools. In addition, the total number of discrepant results generated by each subtyping tool was calculated to evaluate the best-performing subtyping tool among the tested tools. The subtyping tool that generated the fewest discrepancies was considered the best-performing tool for the purposes of this study. Then, the subtypes determined using the best-performing tool based on both *PR/RT* and *pol* regions were subsequently compared against phylogenetic analysis, which is considered the gold standard.

### 2.5. Phylogenetic Analyses of HIV-1 PR/RT and Pol Region Nucleotide Sequences

Subsequently, comparative phylogenetic analyses of the obtained 134 HIV-1 *PR/RT* (2253–3359 in the HXB2 genome) and *pol* (2253–5250 in the HXB2 genome) region nucleotide sequences were carried out. The previously created multiple sequence alignments of both regions were employed to construct two maximum likelihood phylogenetic trees: one tree using *PR/RT* and another tree using *pol* region nucleotide sequences. The comparative evaluation was executed between the subtyping of these regions against phylogenetic clustering. The HIV-1 genotypic subtypes, which were determined using the subtyping tool that generated the fewest discrepancies as previously established, were employed for these comparative analyses to test the specificity of subtype classification. It is noteworthy that REGA 3.0 was established as the subtyping tool that generated the fewest discrepancies. Maximum likelihood phylogenetic trees were constructed using MEGA X software [14]. Regarding the parameter settings, the general time reversible (GTR) model was employed as the nucleotide substitution model, and bootstrap analysis with 1000 replicates was performed to evaluate the reliability of the phylogenetic clustering.

### 2.6. PCR Amplification and Sanger Sequencing of the Near-Full-Length HIV-1 Genome

**Figure 2:**
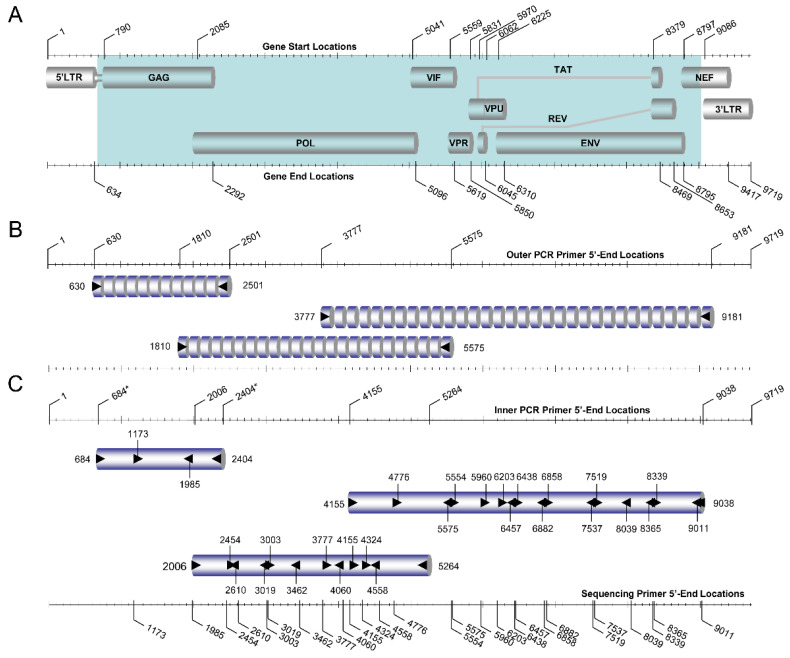
PCR and sequencing design of the near-full-length HIV-1 genome RT–PCR assay. The graphic illustration summarizes the amplification and sequencing of the near-full-length genome of HIV-1 group M subtypes, circulating recombinant forms (CRFs), and recombinants encoding the *gag, pol, vif, vpr, tat, rev, vpu,* and *env* genes. The assay comprises three overlapping amplicons spanning the whole genome amplified via pol RT–PCR assay (2253–5250 in the HXB2 genome) [13], gag RT–PCR assay (790–2292 in the HXB2 genome), and env RT–PCR assay (5041–8795 in the HXB2 genome). (**A**) The top part of the schematic depicts the overall genetic organization of HIV-1 with respect to the HXB2 genome (GenBank accession number K03455), where each major genetic region is represented by labeled gray cylindrical figures. The numbers above and below the diagram show the corresponding beginning and end of these genetic regions indicated by the HXB2 genome. The shaded section, in bright blue in the HIV-1 gene map, denotes the region of amplification in this PCR-based assay of the near-full-length genome of HIV-1. (**B**) The middle part of the schematic presents the primary RT–PCR design for the region of amplification. The numbers on the sides of the three intermittent cylindrical figures illustrating the RT–PCR products of the three overlapping assays represent the corresponding forward and reverse PCR primers. The orientation of the primary RT–PCR primers is indicated with black arrows. The corresponding 5′-end-primer-binding positions of the forward and reverse PCR primers in the primary RT–PCR design are denoted above the schematic in accordance with HXB2 numbering. (**C**) The bottom part of the schematic shows the secondary nested PCR step and the sequencing design for the region of amplification. The numbers on the sides of the three solid cylindrical figures, which represent the secondary nested PCR products of the three overlapping assays, represent the corresponding forward and reverse PCR primers. The orientation of the secondary nested PCR primers is indicated with black arrows. The corresponding 5′-end-primer-binding positions of the forward and reverse PCR primers of the secondary nested PCR design are denoted above the schematic in accordance with HXB2 numbering. The numbers above and below the solid cylindrical figures indicate the forward and reverse sequencing primers, respectively, where the orientation of the sequencing primers is also shown with the labeled black arrows. The numbers below the schematic represent the corresponding 5′-end-primer-binding positions of the sequencing primers in accordance with HXB2 numbering.

For the purpose of identifying the most accurate HIV-1 genotypic subtypes within the limitations imposed by the subtyping tools, the near-full-length HIV-1 genomes (790–8795 in the HXB2 genome) of the 38 ‘discrepant samples’ encoding the *gag, pol, vif, vpr, tat, rev, vpu,* and *env* genes were amplified and sequenced using an in-house-developed comprehensive near-full-length HIV-1 genome RT–PCR assay (Figure 2). The main aims were to delineate the accuracy of phylogenies derived based on HIV-1 *PR/RT* and *pol* region sequences compared to near-full-length HIV-1 genome sequences and to elucidate the genetic nature of the sequences giving rise to discrepancies. Henceforth, the near-full-length HIV-1 genome RT–PCR assay consisting of three overlapping amplicons spanning the whole genome amplified via a previously described HIV-1 pol RT–PCR assay (2253–5250 in the HXB2 genome) [13], HIV-1 gag RT–PCR assay (790–2292 in the HXB2 genome), and HIV-1 env RT–PCR assay (5041–8795 in the HXB2 genome) was adopted. All three PCR assays employed HIV-1-specific PCR and sequencing primers targeting various HIV-1 group M subtypes, CRFs, and recombinants, with the addition of wobbles to any loci that were characterized by more than one nucleotide in the reference dataset of HIV-1 strains. All of the PCR and sequencing primers employed for the near-full-length HIV-1 genome RT–PCR assay are listed in Table 2.

**Figure 3:**
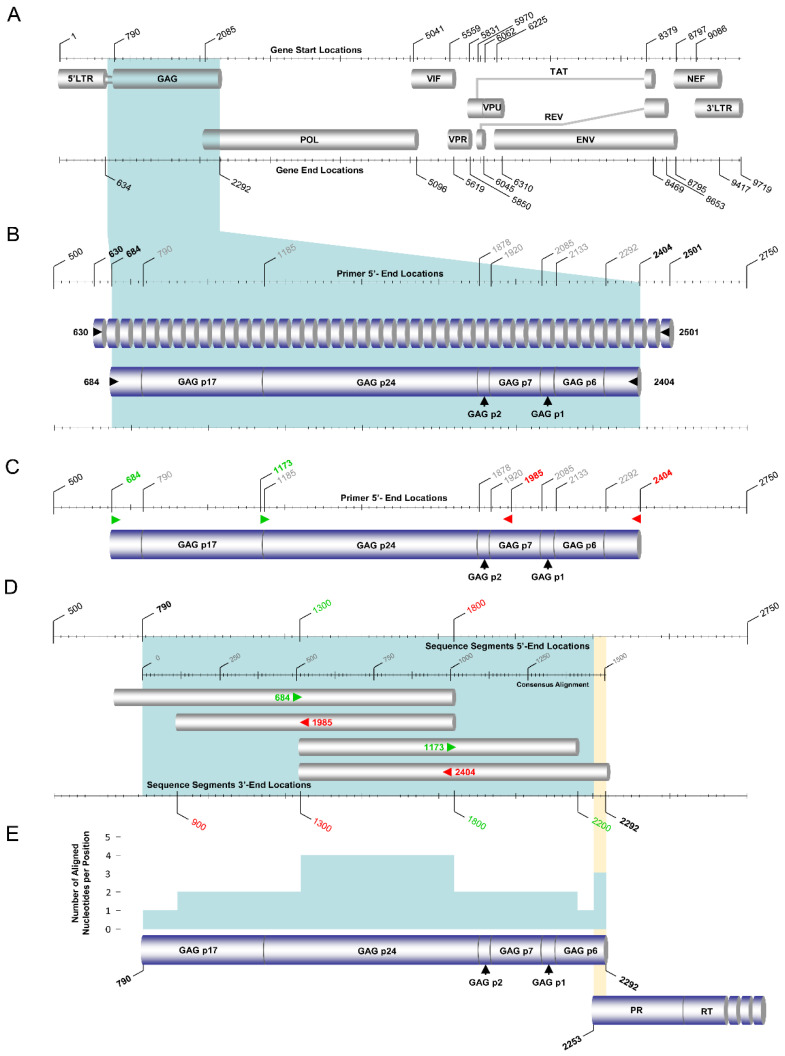
The PCR and sequencing design of the gag RT–PCR assay for the amplification and sequencing of the *gag* (790 to 2292 in the HXB2 genome) region of HIV-1 group M subtypes, circulating recombinant forms (CRFs) and recombinants. (**A**) The top part of the schematic depicts the overall genetic organization of HIV-1 with respect to the HXB2 genome (GenBank accession number K03455), where each major genetic region is represented by labeled gray cylindrical figures. The numbers above and below the diagram show the corresponding beginning and end of these genetic regions indicated by the HXB2 genome. The shaded section in bright blue in the HIV-1 gene map denotes the region of amplification in this gag RT–PCR assay of HIV-1. (**B**) The second part of the schematic presents the primary RT–PCR and the secondary nested PCR design for the region of amplification. The numbers above the diagram in gray correspond to the beginning and end of the genes within the region of amplification (*p17, p24, p2, p7, p1, p6*) with respect to the HXB2 genome. The intermittent and solid cylindrical figures represent the primary RT–PCR and secondary nested PCR products, respectively. The forward and reverse primary (630 and 2501) and secondary (684 and 2404) PCR primers are indicated on the sides of the cylindrical figures. The orientation of the PCR primers is indicated with black arrows. The 5′-end-primer-binding positions of these PCR primers are indicated with the corresponding numbers above the diagram in bold black font, in accordance with HXB2 numbering. (**C**) The solid cylindrical figure indicates the final amplicon resulting from the secondary nested PCR, consisting of the entire *gag* region. The 5′-end-primer-binding positions of forward and reverse sequencing primers are indicated with the corresponding numbers above the diagram in green and red, respectively, in accordance with HXB2 numbering. The orientation of the sequencing primers is also shown with the labeled green and red arrows. The numbers above the diagram in gray correspond to the beginning and end of the genes within the region of amplification (*p17, p24, p2, p7, p1, p6*) with respect to the HXB2 genome. (**D**) The gray cylindrical figures represent the sequencing primer amplicons acquired from labeled sequencing primers, and the orientation of these sequencing primers is indicated with the corresponding arrows. The numbers above and below the diagram indicate the corresponding beginning and end of the sequencing primer amplicons in accordance with HXB2 numbering. (**E**) The top solid cylindrical figure indicates the resulting aligned DNA sequence composed of all the partial sequencing primer amplicons (corresponding to nucleotides 790 to 2292 in the HXB2 genome). This final aligned DNA sequence contains the entire *gag* region. The graph above the resulting aligned DNA sequence shows the number of aligned nucleotides per position when all the partial sequencing primer amplicons are combined. The bottom cylindrical figure indicates the 5′ end of the resulting aligned DNA sequence from the pol RT–PCR assay and the shaded section in bright yellow corresponds to the overlapping region between the final amplicons of the gag and pol RT–PCR assays.

Specifically, the *gag* region (790–2292 in the HXB2 genome) sequences of the ‘discrepant samples’ were amplified using the HIV-1 gag RT–PCR assay (Figure 3). Touchdown PCR was adopted for maximal specificity during annealing steps. For the primary RT–PCR and the secondary nested PCR steps of this assay, the SuperScript™ IV One-Step RT–PCR System (ThermoFisher Scientific, Waltham, MA, USA, 12594025) and the Platinum™ Hot Start PCR 2× Master Mix (ThermoFisher Scientific, Waltham, MA, USA, 13000012) were used, respectively. The primers used for the primary RT–PCR step were 630 and 2501, and those for the secondary nested PCR were 684 and 2404 (Figure 3B). As such, purified HIV-1 plasma RNA was used in the primary RT–PCR step to amplify a 1872 bp product, which was later used in the secondary nested PCR step to amplify a 1721 bp final product.

Initially, to disrupt the secondary structure, 10 μL of HIV-1 RNA from each discrepant sample was incubated at 70 °C for 20 s. The HIV-1 RNA was then immediately placed on ice to stabilize the RNA and incubated for 1 min. Subsequently, 1 μL of each of the primary RT–PCR primers with a concentration of 20 pmol, 0.5 μL of SuperScript IV RT Mix, and 25 μL of 2× Platinum SuperFi RT–PCR Master Mix from the SuperScript™ IV One-Step RT–PCR System as well as 12.5 μL of nuclease-free water, were added to achieve a final volume of 50 μL. The 50 μL reaction mixture was subjected to primary RT–PCR. Upon completion of the PCR, 5 μL of the primary RT–PCR product was employed for the secondary nested PCR step. One microliter of each of the secondary nested PCR primers with a concentration of 20 pmol, 25 μL of Platinum™ Hot Start PCR 2X Master Mix, and 18 μL of nuclease-free water was added to achieve a final volume of 50 μL. PCR amplification was carried out in a SimpliAmp™ Thermal Cycler (Life Technologies, Carlsbad, CA, USA). The PCR thermal cycling conditions of the gag RT–PCR assay are summarized in Table 3. The 1721 bp final product of the secondary nested PCR of the gag RT–PCR assay was subsequently sent to Macrogen Europe (https://dna.macrogen-europe.com/eng/, accessed on 12 September 2022) for purification and Sanger sequencing. The sequencing design of the HIV-1 gag RT–PCR assay involved four sequencing primers: 684, 1173, 1985, and 2404 (Figure 3C,D). The four sequencing primers provided approximately three-primer coverage, ranging from one to four primers at each nucleotide position to achieve reliable sequencing (Figure 3E).

**Figure 4:**
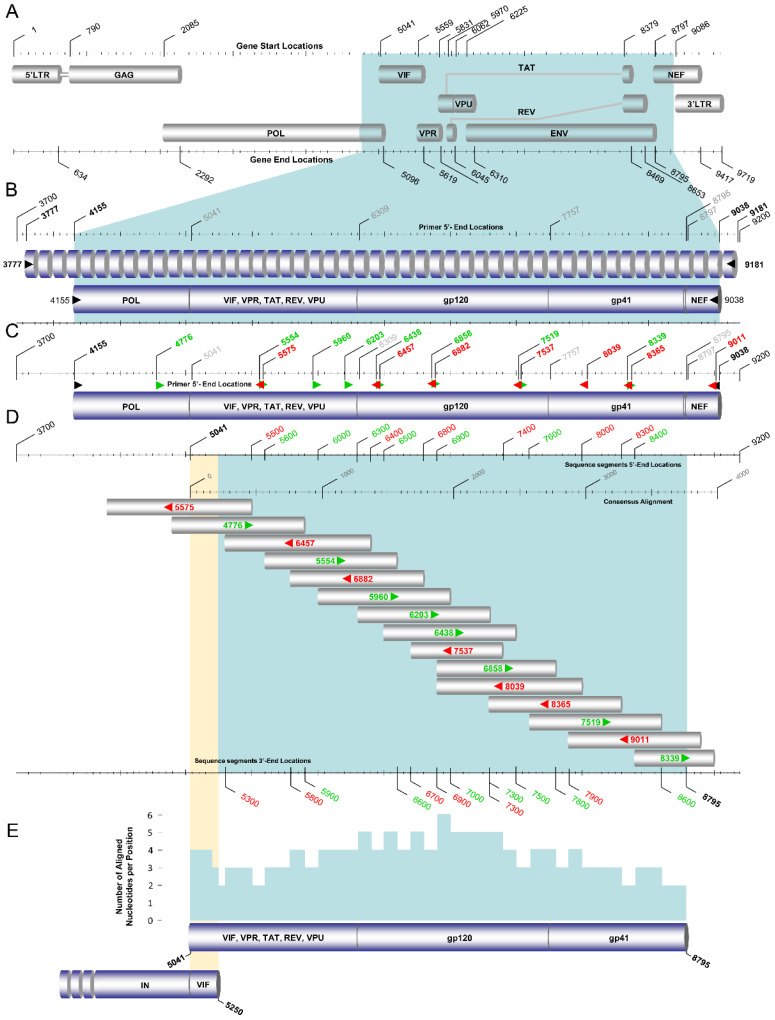
The PCR and sequencing design of the env RT–PCR assay for the amplification and sequencing of the *vif, vpr, tat, rev, vpu,* and *env* (5041 to 8795 in the HXB2 genome) regions of HIV-1 group M subtypes, circulating recombinant forms (CRFs) and recombinants. (**A**) The top part of the schematic depicts the overall genetic organization of HIV-1 with respect to the HXB2 genome (GenBank accession number K03455), where each major genetic region is represented by labeled gray cylindrical figures. The numbers above and below the diagram show the corresponding beginning and end of these genetic regions indicated by the HXB2 genome. The shaded section, in bright blue in the HIV-1 gene map, denotes the region of amplification in this env RT–PCR assay of HIV-1. (**B**) The second part of the schematic presents the primary RT–PCR and the secondary nested PCR design for the region of amplification. The numbers above the diagram in gray correspond to the beginning and end of the genes within the region of amplification (*vif, gp120, gp41, nef*) with respect to the HXB2 genome. The intermittent and solid cylindrical figures represent the primary RT–PCR and secondary nested PCR products, respectively. The forward and reverse primary (3777 and 9181) and secondary (4155 and 9038) PCR primers are indicated on the sides of the cylindrical figures. The orientation of the PCR primers is indicated with black arrows. The 5′-end-primer-binding positions of these PCR primers are indicated with the corresponding numbers above the diagram in bold black color, in accordance with HXB2 numbering. (**C**) The solid cylindrical figure indicates the final amplicon resulting from the secondary nested PCR, consisting of the entire *vif, vpr, tat, rev, vpu, env,* and 5′ end of the *nef* regions. The 5′-end-primer-binding positions of forward and reverse sequencing primers are indicated with the corresponding numbers above the diagram in green and red, respectively, in accordance with HXB2 numbering, while the bold black numbers indicate the secondary nested PCR primers. The orientation of the sequencing primers is also shown with the labeled green and red arrows. The numbers above the diagram in gray correspond to the beginning and end of the genes within the region of amplification (*vif, gp120, gp41, nef*) with respect to the HXB2 genome. (**D**) The gray cylindrical figures represent the sequencing primer amplicons acquired from labeled sequencing primers, and the orientation of these sequencing primers is indicated with the corresponding arrows. The numbers above and below the diagram indicate the corresponding beginning and end of the sequencing primer amplicons in accordance with HXB2 numbering. (**E**) The top solid cylindrical figure indicates the resulting aligned DNA sequence composed of all the partial sequencing primer amplicons (corresponding to nucleotides 5041 to 8795 in the HXB2 genome). This final aligned DNA sequence contains the entire *vif, vpr, tat, rev, vpu,* and *env* regions. The graph above the resulting aligned DNA sequence demonstrates the number of aligned nucleotides per position when all the partial sequencing primer amplicons are combined. The bottom cylindrical figure indicates the 3′ end of the resulting aligned DNA sequence from the pol RT–PCR assay and the shaded section in bright yellow represents the overlapping region between the final amplicons of the pol and env RT–PCR assays.

Following the amplification of the *gag* region, the *env* region (5041–8795 in the HXB2 genome) sequences of the ‘discrepant samples’ were amplified using the HIV-1 env RT–PCR assay (Figure 4). Similar to the gag and pol RT–PCR assays, touchdown PCR was also adopted for maximal specificity during the annealing step of primary RT–PCR in this assay. For the primary RT–PCR and the secondary nested PCR steps, the SuperScript™ IV One-Step RT–PCR System (ThermoFisher Scientific, Waltham, MA, USA, 12594025) and Platinum™ SuperFi II PCR Master Mix (ThermoFisher Scientific, Waltham, MA, USA, 12368010) were used, respectively. The primary RT–PCR product of the env RT–PCR assay is over 5 kb. Therefore, Platinum™ SuperFi II PCR Master Mix was chosen for this reaction due to its ability to amplify products up to 20 kb, while Platinum™ Hot Start PCR 2× Master Mix is limited to the amplification of products up to 5 kb. The primers used for primary RT–PCR were 3777 and 9181, and those for the secondary nested PCR were 4155 and 9038 (Figure 4B). As such, purified HIV-1 plasma RNA was used in the primary RT–PCR step to amplify a 5405 bp product, which was then used for secondary nested PCR to amplify a 4884 bp final product.

The unfolding and stabilization of HIV-1 plasma RNA were performed as explained above in the gag RT–PCR assay. For the protocol of the env RT–PCR assay, slightly different PCR primer concentrations were employed to yield the optimal product concentration, which was considered necessary due to high genetic variation in the *env* region among different HIV-1 strains. Following the incubation of the HIV-1 plasma RNA on ice, 1 μL of each of the primary RT–PCR primers with a concentration of 80 pmol, 0.5 μL of SuperScript IV RT Mix, and 25 μL of 2× Platinum SuperFi RT–PCR Master Mix from the SuperScript IV One-Step RT–PCR System and 12.5 μL of nuclease-free water were added to achieve a final volume of 50 μL. The 50 μL reaction mixture was subjected to primary RT–PCR. Upon the completion of PCR, 5 μL of the primary RT–PCR product was employed for the secondary nested PCR. One microliter of the forward secondary nested PCR primer with a concentration of 40 pmol, 1 μL of the reverse secondary nested PCR primer with a concentration of 80 pmol, 25 μL of Platinum™ SuperFi II PCR Master Mix, and 18 μL of nuclease-free water were added to achieve a final volume of 50 μL. PCR amplification was again carried out in a SimpliAmp™ Thermal Cycler (Life Technologies, Carlsbad, CA, USA). The PCR thermal cycling conditions of the env RT–PCR assay are summarized in Table 4. The purification and Sanger sequencing of the 4884 bp final product of the secondary nested PCR step of the env RT–PCR assay were also performed by Macrogen Europe (https://dna.macrogen-europe.com/eng/, accessed on 12 September 2022). The sequencing design of the HIV-1 env RT–PCR assay involved 15 sequencing primers: 4776, 5554, 5575, 5960, 6203, 6438, 6457, 6858, 6882, 7519, 7537, 8039, 8339, 8365, and 9011 (Figure 4C,D). The 15 sequencing primers provided approximately four-primer coverage, ranging from two to six primers at each nucleotide position to achieve reliable sequencing (Figure 4E).

**Figure 5:**
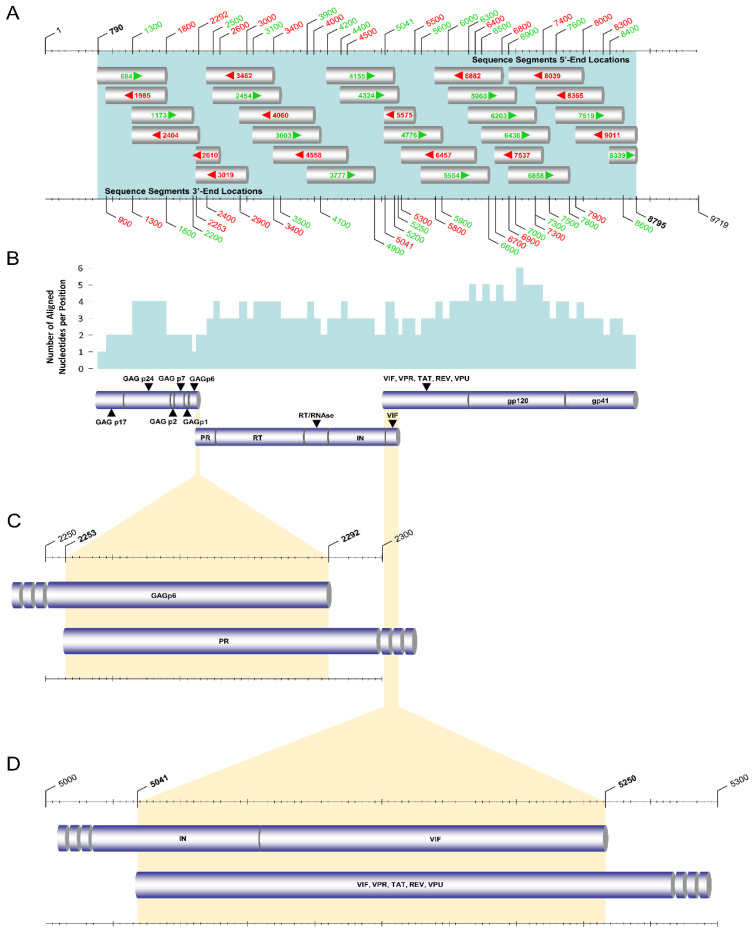
The sequencing design of the near-full-length HIV-1 genome RT–PCR assay. The graphic illustration summarizes the sequencing of the near-full-length genome of HIV-1 group M subtypes, circulating recombinant forms (CRFs), and recombinants and depicts the overlapping regions between the three final amplicons of the gag, pol, and env RT–PCR assays. (**A**) The gray cylindrical figures represent the sequence segments acquired from labeled sequencing primers, and the orientation of these sequencing primers are indicated with the corresponding green and red arrows. The numbers above and below the diagram indicate the corresponding beginning and end of the sequence segments in accordance with HXB2 numbering. (**B**) The three solid cylindrical figures indicate the resulting aligned DNA sequences composed of all the partial sequence segments (corresponding to nucleotides 790 to 8795 in the HXB2 genome) derived from the overlapping gag, pol, and env RT–PCR assays. The final aligned DNA sequence contains the near-full-length HIV-1 genome, encoding the *gag, pol, vif, vpr, tat, rev, vpu,* and *env* genes. The graph above the resulting aligned DNA sequences demonstrates the number of aligned nucleotides per position when all the partial sequence segments are combined. The shaded sections in bright yellow represent the overlapping regions between the final amplicons of the gag, pol, and env RT–PCR assays. (**C**) The schematic shows a magnified view of the overlapping region between the final amplicons of the gag and pol RT–PCR assays. The top cylindrical figure indicates the 3′ end of the resulting aligned DNA sequence from the gag RT–PCR assay. The bottom cylindrical figure indicates the 5′ end of the resulting aligned DNA sequence from the pol RT–PCR assay. The bold black numbers above the graph delineate the overlapping region in accordance with HXB2 numbering. (**D**) The schematic shows a magnified view of the overlapping region between the final amplicons of the pol and env RT–PCR assays. The top cylindrical figure indicates the 3′ end of the resulting aligned DNA sequence from the pol RT–PCR assay. The bottom cylindrical figure indicates the 5′ end of the resulting aligned DNA sequence from the env RT–PCR assay. The bold black numbers above the graph delineate the overlapping region in accordance with HXB2 numbering.

Overall, for the sequencing of the final products amplified in the three overlapping RT–PCR assays, a total of 29 sequencing primers were utilized to obtain the near-full-length HIV-1 genome nucleotide sequences (Figure 5A). Once the sequencing primer amplicons were received from Macrogen Europe, they were input into Geneious^®^ 11.1.4 software (https://www.geneious.com, accessed on 12 September 2022) and aligned against the HXB2 genome (GenBank accession number K03455). The qualitative analyses of the sequencing information and the success of each sequencing primer amplicon were performed according to a previously published protocol [13]. When all sequencing primer amplicons were aligned, the mean number of aligned nucleotides per position was found to be approximately three, ranging from one to six at each nucleotide position (Figure 5B). Consequently, to obtain the consensus near-full-length HIV-1 genome sequences of the 38 ‘discrepant samples’, the final HIV-1 *gag* and *env* region amplicons were aligned with the previously obtained HIV-1 *pol* region amplicons. Accordingly, there was a 40-nucleotide overlapping region between the gag and pol RT–PCR assays and a 210-nucleotide overlapping region between the pol and env RT–PCR assays (Figure 5C).

### 2.7. Subtyping and Phylogenetic Analyses of Near-Full-Length HIV-1 Genome Nucleotide Sequences

The 38 near-full-length HIV-1 genome (790–8795 in the HXB2 genome) nucleotide sequences obtained through Sanger sequencing were analyzed for HIV-1 genotypic subtypes using the subtyping tool that generated the fewest discrepancies, which was previously established to be REGA 3.0. The HIV-1 genotypic subtypes of each of the ‘discrepant samples’ determined based on near-full-length genome sequences were retrospectively compared to the HIV-1 genotypic subtypes determined based on *PR/RT* and *pol* region sequences using each of the six subtyping tools. The analyses indicated whether the ‘discrepant samples’ were either pure group M subtypes, CRFs, or uncharacterized recombinant HIV-1 strains and if they were correctly defined by any of the subtyping tools based on partial HIV-1 sequences.

Finally, a comparative phylogenetic analysis of the obtained near-full-length HIV-1 genome (790–8795 in the HXB2 genome) nucleotide sequences of the 38 ‘discrepant samples’ was carried out. The phylogenetic analyses were conducted against a reference dataset of HIV-1 subtypes and CRFs using the RIP Alignment 2017 downloaded from the Los Alamos HIV Sequence Database (https://www.hiv.lanl.gov/content/sequence/NEWALIGN/align.html, accessed on 12 September 2022) for the confirmation of genotypic subtypes against the gold standard. Initially, a multiple sequence alignment of the near-full-length HIV-1 genome sequences was constructed using the ClustalW algorithm with MEGA X software [14]. Multiple sequence alignment was then employed for maximum likelihood phylogenetic tree construction in MEGA X software. Regarding the parameter settings, the GTR model was employed as the nucleotide substitution model, and bootstrap analysis with 1000 replicates was performed to evaluate the reliability of the phylogenetic clustering. The comparative evaluation was executed between the subtyping of the near-full-length HIV-1 genome sequences against phylogenetic clustering to investigate the specificity of subtype classification.

## 3. Results

### 3.1. Clinical, Epidemiological and Behavioral Information of the Study Participants

We included 134 consenting newly diagnosed or chronically HIV-1-infected patients in this study. We collected data from previously published research conducted by our laboratory focused on the development of an HIV-1 genotypic drug resistance assay [13]. A total of 134 out of 144 HIV-1-infected study participants included in the previously published research project were included in this study. Among the remaining 10 patient samples, three samples were excluded from this study due to being PCR negative, and seven samples were excluded due to failure in the sequencing of the HIV-1 *env* region (5041–8795 in the HXB2 genome) because of low-quality reads. The collection dates of the 134 patient samples used in this study ranged from 9 March 2017 to 1 August 2019. Among the 134 HIV-1-infected study participants from whom blood samples were collected, 114 were newly diagnosed, while 20 were chronic patients. None of the newly diagnosed study participants were receiving antiretroviral therapy. However, 10 of the 20 chronic study participants were on cART, while the remaining 10 were not.

The epidemiological information gathered from the questionnaires showed that the cohort mostly comprised males (*n* = 111, 82.8%). The age of the study participants ranged from 24 to 76, with an average age of 41.4, while the most common age group was ‘31–40′ (*n* = 54, 40.3%). According to the behavioral information collected, the most common route of infection reported was ‘men who have sex with men’ (MSM) (*n* = 62, 46.3%), followed by ‘heterosexual contact’ (HC) (*n* = 44, 32.8%) and ‘homo/bisexual contact’ (HBC) (*n* = 17, 12.7%). There were also two study participants who reported ‘injecting drug user’ (IDU) (*n* = 2, 1.5%) and one study participant who reported ‘blood transfusion’ (TR) (*n* = 1, 0.7%) as the route of infection. Moreover, three study participants reported either HC or TR (*n* = 3, 2.2%), two study participants reported either HC or IDU (*n* = 2, 1.5%), one study participant reported either MSM or IDU (*n* = 1, 0.7%), and two study participants reported either HC or ‘originating from high-prevalence country’ (OHPC) (*n* = 2, 1.5%). The most common country of origin in the cohort was Cyprus (*n* = 72, 53.7%), followed by Cameroon (*n* = 16, 11.9%), Romania (*n* = 11, 8.2%), Greece (*n* = 8, 6.0%), the United Kingdom (U.K.) (*n* = 6, 4.5%), Bulgaria (*n* = 6, 4.5%), Nigeria (*n* = 3, 2.2%) and Russia (*n* = 2, 1.5%). There was also one HIV-1-infected patient originating from each of the following countries: Congo, Egypt, Hungary, Indonesia, Italy, Ivory Coast, Lebanon, the Philippines, Sri Lanka, and Ukraine (*n* = 1, 0.7% each). Most of the HIV-1-infected patients included in this cohort reported being infected in Cyprus (*n* = 82, 61.2%), followed by Cameroon (*n* = 12, 12.7%), Greece (*n* = 6, 4.5%), the U.K. (*n* = 4, 3.0%), Bulgaria (*n* = 2, 1.5%), Romania (*n* = 2, 1.5%) and Nigeria (*n* = 2, 1.5%). There was one HIV-1-infected patient who reported being infected in each of the following countries: Holland, India, Ivory Coast, Russia, Sri Lanka, Spain, Ukraine, Venezuela, and Zimbabwe (*n* = 1, 0.7% each). There were also three study participants who reported their country of infection as either Cyprus or Austria (*n* = 1, 0.7%), Cyprus or U.K. (*n* = 1, 0.7%), and Europe (*n* = 1, 0.7%). Additionally, there were seven study participants who declared their country of infection unknown (*n* = 7, 5.2%). The most common district of residency of the cohort was identified as Nicosia (*n* = 64, 47.8%), which is the capital of Cyprus, followed by Limassol (*n* = 28, 20.9%), Larnaca (*n* = 21, 15.7%), Pafos (*n* = 15, 11.2%) and Famagusta (*n* = 6, 4.5%). All the blood samples collected from the HIV-1-infected patients included in this study had viral loads equal to or above 10^3^ RNA copies/mL plasma, in accordance with the inclusion criteria. The lowest and highest viral loads in the cohort were 1080 RNA copies/mL plasma and 5,320,000 RNA copies/mL plasma, respectively.

### 3.2. Discrepancies among the HIV-1 Subtyping Tools

Initially, the *pol* region (2253–5250 in the HXB2 genome) sequences encoding the *PR, RT, IN,* and partial *vif* genes of the 134 HIV-1 viral genomes were successfully amplified and sequenced according to a previously published touchdown HIV-1 pol RT–PCR assay [13]. Thereafter, the HIV-1 *PR/RT* (2253–3359 in the HXB2 genome) region sequences were extracted from the HIV-1 *pol* (2253–5250 in the HXB2 genome) region sequences using AliView software [15]. Table S1 of the Supplementary Material provides the HIV-1 genotypic subtypes determined based on the *PR/RT* and *pol* region sequences of all 134 samples using six different subtyping tools. With the aim of identifying ‘discrepant samples’, the determined subtypes were analyzed for any disagreements among the different subtyping tools according to the abovementioned specifications (Table S1). Accordingly, 53 of the 134 patient samples (*n* = 53, 39.6%) showed disagreements in the subtyping of the *PR/RT* region among the different subtyping tools. These samples were considered to be ‘discrepant in the *PR/RT* region’. However, 38 of the 134 patient samples (*n* = 38, 28.4%) presented disagreements in the subtyping of the *pol* region among the different subtyping tools and, hence, were considered to be ‘discrepant in the *pol* region’. Among the 53 samples identified as ‘discrepant in the *PR/RT* region’, 19 samples showed discrepancies only in the *PR/RT* region. Moreover, among the 38 samples identified as ‘discrepant in the *pol* region’, four samples showed discrepancies in only the *pol* region. The remaining 34 samples showed discrepancies in both the *PR/RT* and *pol* regions. Thirty-eight patient samples that showed disagreements in both the *PR/RT* and *pol* regions or only in the *pol* region were assigned the final designation of a ‘discrepant sample’. The HIV-1 genotypic subtypes determined based on the *PR/RT* and *pol* region sequences of the 38 ‘discrepant samples’ and the disagreements identified among the six different subtyping tools are summarized in Table 5.

The results also showed that, collectively in both regions, the lowest number of discrepancies was generated by REGA 3.0 (*n* = 17), followed by Stanford (*n* = 18), COMET 2.3 (*n* = 24), SCUEAL (*n* = 26), jpHMM (*n* = 38) and Geno2pheno (*n* = 40). Therefore, REGA 3.0 was considered the best-performing tool among the tested tools for the purposes of this study. Henceforth, the subtypes generated by REGA 3.0 determined based on the two regions were employed for the following comparative phylogenetic analyses.

With respect to the HIV-1 genotypic subtypes of the 134 samples determined based on the *PR/RT* (2253–3359 in the HXB2 genome) region sequences (Table S1), subtype B was identified as the predominant subtype in Cyprus (*n* = 36, 26.9%). The second and third predominant subtypes were sub-subtype A1 (*n* = 33, 24.6%) and CRF02_AG (*n* = 22, 16.4%), respectively. The following subtypes were found to be scarcer: F1 (*n* = 10, 7.5%); G (*n* = 4, 3.0%); C (*n* = 3, 2.2%); CRF01_AE (*n* = 3, 2.2%); and CRF06_cpx (*n* = 3, 2.2%). Additionally, one of each of sub-subtype A2, subtype D, CRF03_AB, CRF04_cpx and CRF35_AD was identified (*n* = 1, 0.7% each). It should be highlighted that the remaining 15 samples were identified as uncharacterized HIV-1 recombinants (*n* = 15, 11.2%). Additionally, according to the HIV-1 genotypic subtypes determined based on the *pol* (2253–5250 in the HXB2 genome) region sequences (Table S1), sub-subtype A1 was identified as the predominant subtype in Cyprus (*n* = 32, 23.9%). The second and third predominant subtypes were subtype B (*n* = 31, 23.1%) and CRF02_AG (*n* = 20, 14.9%), respectively. Similarly, the fourth predominant subtype was again identified as sub-subtype F1 (*n* = 9, 6.7%), followed by subtype C (*n* = 3, 2.2%), CRF01_AE (*n* = 3, 2.2%) and CRF06_cpx (*n* = 3, 2.2%). Each of the following subtypes was identified once: A2; D; G; CRF03_AB; CRF04_cpx; and CRF18_cpx (*n* = 1, 0.7% each). A comparison of the subtypes determined based on the *pol* region with the subtypes determined based on the *PR/RT* region revealed an 8.9% increase in the prevalence of uncharacterized HIV-1 recombinants, from 11.2% to 20.1% (*n* = 27, 20.1%). It is important to mention that when the *pol* region sequences of the 38 ‘discrepant samples’ were analyzed using REGA 3.0, four were identified to belong to pure group M subtypes (*n* = 4, 10.5%), eight were CRFs (*n* = 8, 21.1%) and the vast majority were determined as uncharacterized recombinant HIV-1 strains (*n* = 26, 68.4%).

### 3.3. Comparative Phylogenetic Analyses

**Figure 6:**
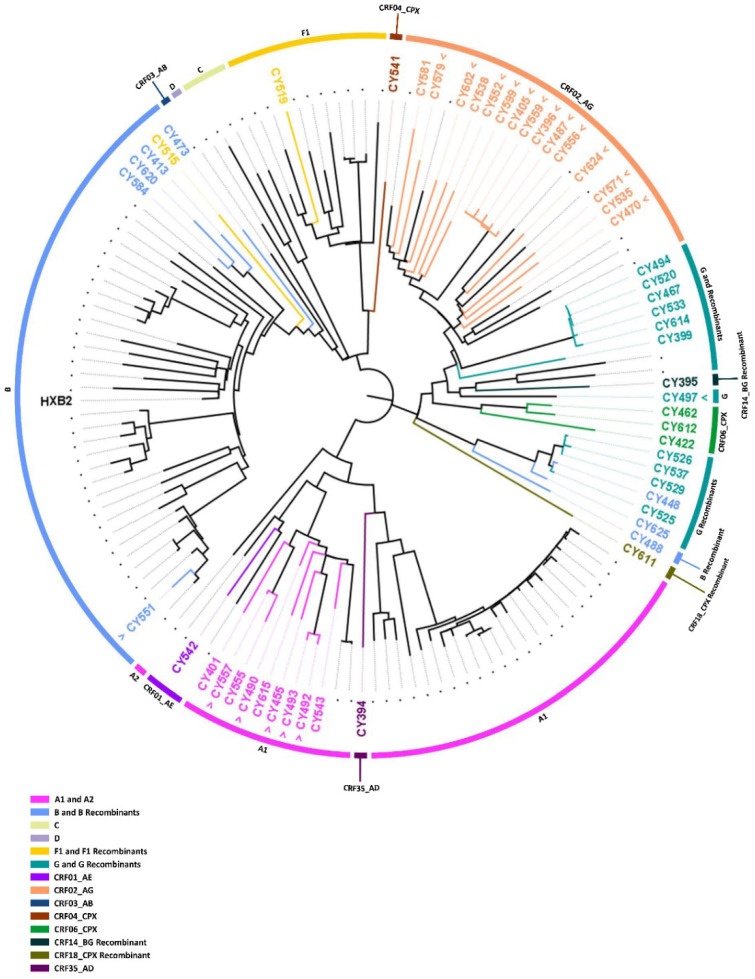
Maximum likelihood phylogenetic tree analyses of the *‘PR/RT’* region sequences (corresponding to nucleotides 2253 to 3359 in the HXB2 genome) of 134 HIV-1-infected patient samples from Cyprus used in this study. The 34 samples that presented discrepancies in the subtyping of both the *‘PR/RT’* and *‘POL’* regions among the different genotypic subtyping tools tested are indicated using their unique laboratory identification numbers, in which the prefix CY is followed by a number denoting the laboratory code. The 19 samples that presented discrepancies in the subtyping of only the *‘PR/RT’* region are indicated with a greater-than sign next to their unique laboratory identification numbers, while the samples that did not present any discrepancies in the subtyping of the *‘PR/RT’* region are indicated with a black dot. The HIV-1 clades are color coded at the periphery of the maximum likelihood tree based on the HIV-1 genotypic subtypes of the *‘PR/RT’* region sequences determined by the REGA HIV-1 subtyping tool, version 3.0 (http://dbpartners.stanford.edu:8080/RegaSubtyping/stanford-hiv/typingtool/, accessed on 12 September 2022), and the color coding is defined below the tree. The same color coding is employed for the HIV-1 genotypic subtypes of each sample, as determined by the REGA HIV-1 subtyping tool, version 3.0, as well as the corresponding branch in the maximum likelihood tree, for the comparative evaluation of the subtyping of the *‘PR/RT’* region against phylogenetic clustering.

**Figure 7:**
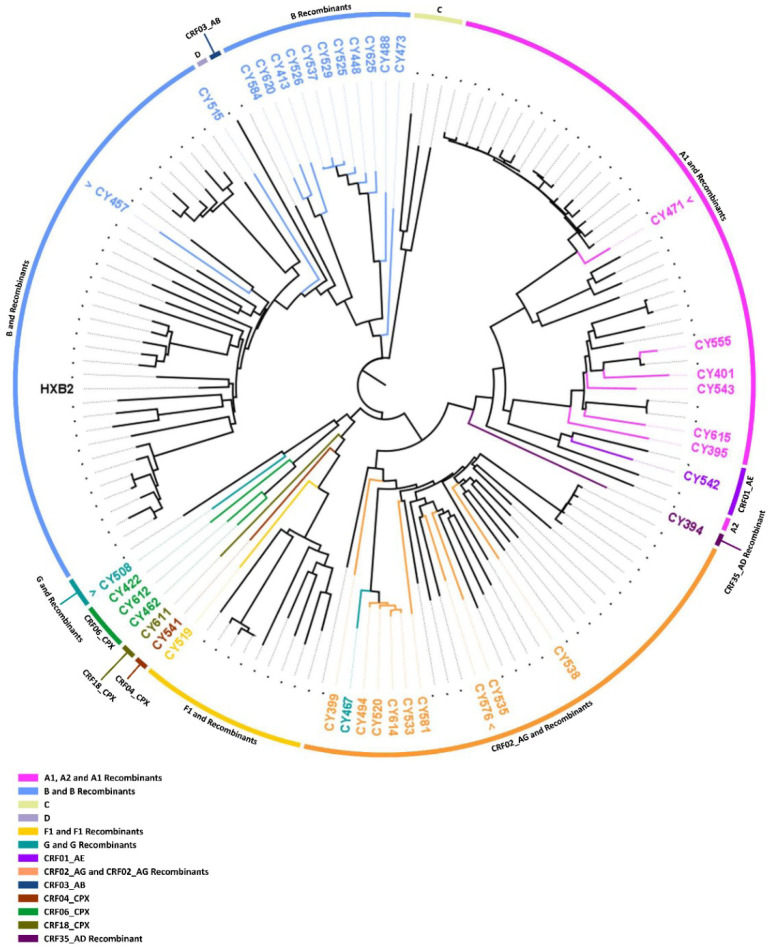
Maximum likelihood phylogenetic tree analyses of the *‘POL’* region sequences (corresponding to nucleotides 2253 to 5250 in the HXB2 genome) of 134 HIV-1-infected patient samples from Cyprus used in this study. The 34 samples that presented discrepancies in the subtyping of both the *‘PR/RT’* and *‘POL’* regions among the different genotypic subtyping tools tested are indicated using their unique laboratory identification numbers, in which the prefix CY is followed by a number denoting the laboratory code. The four samples that presented discrepancies in the subtyping of only the *‘POL’* region are indicated with a greater-than sign next to their unique laboratory identification numbers, while the samples that did not present any discrepancies in the subtyping of the *‘POL’* region are indicated with a black dot. The HIV-1 clades are color coded at the periphery of the maximum likelihood tree based on the HIV-1 genotypic subtypes of the *‘POL’* region sequences determined by the REGA HIV-1 subtyping tool, version 3.0 (http://dbpartners.stanford.edu:8080/RegaSubtyping/stanford-hiv/typingtool/, accessed on 12 September 2022), and the color coding is defined below the tree. The same color coding is employed for the HIV-1 genotypic subtypes of each sample, as determined by the REGA HIV-1 subtyping tool, version 3.0, as well as the corresponding branch in the maximum likelihood tree, for comparative evaluation of the subtyping of the *‘POL’* region against phylogenetic clustering.

For the comparative phylogenetic analyses, a maximum likelihood tree was constructed using HIV-1 *PR/RT* (2253–3359 in the HXB2 genome) region sequences (Figure 6) and *pol* (2253–5250 in the HXB2 genome) region sequences (Figure 7) derived from 134 HIV-1-infected study participants. The HIV-1 genotypic subtypes determined using REGA 3.0 were employed for these comparative analyses, as REGA 3.0 was identified as the subtyping tool that generated the fewest discrepancies. The comparative phylogenetic analyses of the *PR/RT* region sequences illustrated that the HIV-1 clades were mostly consistent and agreed with the HIV-1 genotypic subtypes determined by REGA 3.0. This can be observed in Figure 6 since, among the 53 samples that were ‘discrepant in the *PR/RT* region’, 50 samples showed consistency in subtyping with the HIV-1 clades that were formed. The samples that presented inconsistency in subtyping compared to phylogenetic clustering can be observed in Figure 6, where the color coding of the periphery of the maximum likelihood tree representing the HIV-1 clade does not match the color coding of the sample name representing the subtype classification. This phenomenon can be explained by the fact that the inconsistent samples were recombinants represented by two subtypes, where the latter subtype explains the disagreement between subtype classification and phylogenetic clustering. In particular, the HIV-1 genotypic subtypes of samples CY515, CY448, and CY625 could be observed to be inconsistent with the HIV-1 clades (Figure 6). Specifically, sample CY515 was identified as “Rec. of F1, B” and showed clustering with samples determined to be subtype B with a bootstrap support value of 30.0%. In addition, samples CY448 and CY625 were identified as “Rec. of B, G”, and they showed clustering with samples determined to be subtype G recombinants. The clade comprising samples CY448 and CY625 was supported by a 100% bootstrap support value; however, sample CY625 clustered away from the other samples, which formed a sub-clade. Thus, even though there were minor inconsistencies between clustering and subtyping, placement within the phylogenetic tree essentially did not deviate from the subtyping results since the recombinant samples contained significant regions of both genotypes.

Similarly, the comparative phylogenetic analyses of the *pol* region sequences showed parallel results with the *PR/RT* region sequences, presenting HIV-1 clades consistent with the HIV-1 genotypic subtypes determined by REGA 3.0. As depicted in Figure 7, 37 out of 38 samples that were ‘discrepant in the *pol* region’ exhibited agreement between subtyping and phylogenetic clustering. The only sample that presented inconsistency was sample CY467. Sample CY467 was identified as “Rec. of G, A1, B”, while it showed clustering with the samples determined to be CRF02_AG and CRF02_AG recombinants. However, sample CY467 showed indications of slight divergence from the clade with which it clustered, which could explain the inconsistency identified or could suggest an incorrectly identified subtype due to the use of a partial sequence. Specifically, the clade comprising sample CY467 was supported by a 100% bootstrap support value; however, sample CY467 clustered away from the other samples, which formed a sub-clade.

### 3.4. Characterization by Near-Full-Length Genome Sequencing

Upon confirming the results of the comparative phylogenetic analyses, the samples identified as ‘discrepant samples’ were selected for near-full-length HIV-1 genome (790–8795 in the HXB2 genome) sequencing. As such, the near-full-length HIV-1 genomes of the 38 ‘discrepant samples’ encoding the *gag, pol, vif, vpr, tat, rev, vpu,* and *env* genes were successfully amplified and sequenced. The obtained sequences were then used to determine the HIV-1 genotypic subtypes using REGA 3.0. The HIV-1 genotypic subtypes determined based on the near-full-length HIV-1 genome sequences of the 38 ‘discrepant samples’ are presented in Table 6. The identified HIV-1 genotypic subtypes based on the HIV-1 near-full-length genome sequences revealed that six of the 38 ‘discrepant samples’ belonged to pure group M subtypes (*n* = 6, 15.8%), eight were CRFs (*n* = 8, 21.1%), and 24 were uncharacterized recombinant HIV-1 strains (*n* = 24, 63.2%). Specifically, sub-subtype A1 (*n* = 5, 13.2%) and subtype B (*n* = 1, 2.6%) were identified among the pure group M subtypes, and CRF02_AG (*n* = 4, 10.5%), CRF06_cpx (*n* = 3, 7.9%) and CRF04_cpx (*n* = 1, 2.6%) were identified among the CRFs. Additionally, among the 24 uncharacterized HIV-1 recombinants, nine were recombinants represented by two subtypes/CRFs (*n* = 9, 23.7%), whereas the remaining 15 were complex recombinants, which are recombinants represented by three or more subtypes/CRFs (*n* = 15, 39.5%).

**Figure 8:**
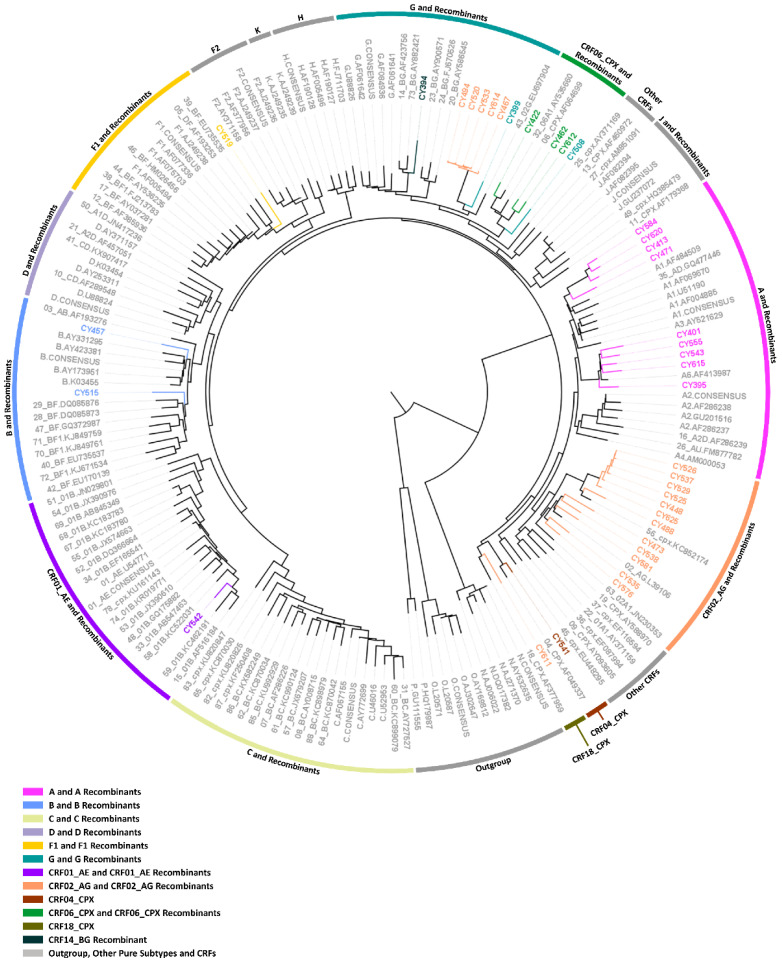
Maximum likelihood phylogenetic tree analyses of the near-full-length HIV-1 genome sequences (corresponding to nucleotides 790 to 8795 in the HXB2 genome) of 38 HIV-1-infected patient samples from Cyprus, which presented discrepancies in both the ‘*PR/RT’* and ‘*POL*’ regions or only in the ‘*POL*’ region. The phylogenetic analyses were conducted against a reference dataset of HIV-1 subtypes and CRFs using RIP Alignment 2017 downloaded from the Los Alamos HIV Sequence Database (https://www.hiv.lanl.gov/content/sequence/NEWALIGN/align.html, accessed on 12 September 2022). The 38 samples that presented discrepancies in the subtyping of both ‘*PR/RT’* and *‘POL’* regions or only the ‘*POL*’ region among the different tested genotypic subtyping tools are indicated using their unique laboratory identification numbers, in which the prefix CY is followed by a number denoting the laboratory code. Each reference sequence is named to display the HIV-1 genotypic subtype, followed by the GenBank accession number, as indicated in gray. The HIV-1 clades are color coded at the periphery of the maximum likelihood tree based on the HIV-1 genotypic subtypes of the near-full-length HIV-1 genome sequences, as provided by the Los Alamos HIV Sequence Database (http://www.hiv.lanl.gov, accessed on 12 September 2022), and the color coding is defined below the tree. The same color coding is employed for the HIV-1 genotypic subtypes of each sample as determined by the REGA HIV-1 subtyping tool, version 3.0 (http://dbpartners.stanford.edu:8080/RegaSubtyping/stanford-hiv/typingtool/, accessed on 12 September 2022), as well as the corresponding branch in the maximum likelihood tree, for the comparative evaluation of the subtyping of the near-full-length HIV-1 genome against the phylogenetic clustering results.

For a final confirmatory comparative phylogenetic analysis, a maximum likelihood tree of the near-full-length HIV-1 genome (790–8795 in the HXB2 genome) sequences of the 38 ‘discrepant samples’ was constructed against a reference dataset of HIV-1 subtypes and CRFs using RIP Alignment 2017 (Figure 8). The comparative phylogenetic analyses demonstrated that the HIV-1 clades were mostly consistent and agreed with the HIV-1 genotypic subtypes determined by REGA 3.0. Consistency between subtype classification and phylogenetic clustering was observed for 30 out of 38 ‘discrepant samples’. The remaining eight samples that showed inconsistency between the subtyping results and the phylogenetic clustering results can be observed in Figure 8, where the color coding at the periphery of the maximum likelihood tree representing the HIV-1 clade does not match the color coding of the sample name representing the subtype classification. As previously noted, the occurrence of these inconsistencies can be explained by the fact that inconsistent samples were recombinants represented by two or more subtypes/CRFs, and one of the latter subtypes thus explained the disagreement between subtype classification and phylogenetic clustering.

Specifically, samples CY394, CY508, CY467, CY494, CY520, CY533, CY614, and CY611 exhibited such inconsistencies between their subtypes assigned by REGA 3.0 and the HIV-1 clades formed on the maximum likelihood tree. First, sample CY394 was identified as “Rec. of 14_BG, A1, D”, and it showed clustering with reference sequences for subtype G and its recombinants. In particular, sample CY394 clustered with CRF14_BG and CRF73_BG with a 100% bootstrap support value. Second, sample CY508 was determined to be “Rec. of G, 06_cpx”, and it showed clustering with reference sequences for CRF06_cpx and CRF06_cpx recombinants with a bootstrap support value of 100%. The five samples, CY467, CY494, CY520, CY533, and CY614, were identified as either “Rec. of 02_AG, G, J”, “Rec. of 02_AG, G, J, A1”, and “Rec. of 02_AG, G, B, J, D”, and they showed clustering with reference sequences for subtype G and its recombinants. Specifically, these samples formed a molecular cluster defined by a genetic distance of less than 0.045 and were supported by a bootstrap support value of 100%. Finally, sample CY611, which was subtyped as “Rec. of 02_AG, F1, A1, G”, showed clustering with the reference sequence of CRF18_cpx with a 99% bootstrap support value. The subtypes constituting this recombinant sample and phylogenetic clustering suggested that it could be a recombinant of CRF18_cpx that was incorrectly subtyped [24]. Analogous to the previous comparative phylogenetic analyses, it has been illustrated that although there are slight inconsistencies between phylogenetic clustering and subtyping, the placement within the phylogenetic tree essentially does not diverge from the subtyping results, since the recombinant samples are represented by significant proportions of more than one genotype.

However, when the phylogenetic tree was analyzed in more detail, it was revealed that four of the five samples identified as A1 (CY401, CY543, CY555, and CY615) formed a clade with the reference sequence for sub-subtype A6 with an 84.3% bootstrap support value. Subsequent bootscan and similarity plot analyses confirmed that these samples belonged to sub-subtype A6. Additionally, the remaining sample identified as A1 (CY471) presented little divergence from the reference sequences for A1 and formed a clade with samples CY413, CY584, and CY620 with an 88.1% bootstrap support value, which was identified as “Rec. of A1, B”. However, it is noteworthy that the sample CY471 clustered away from the other samples in this clade. Follow-up bootscan and similarity plot analyses also confirmed that this sample is “Rec. of A1, B”, with a small fragment of B recombined into a backbone of A1. Likewise, the sample identified as subtype B (CY457) exhibited similar divergence from the reference sequences for subtype B. While the clade was supported by a 93.8% bootstrap support value, sample CY457 clustered away from the references sequences for pure subtype B. Bootscan and similarity plot analyses classified the sample as “Rec. of B, A1”, with a small fragment of A1 recombined into a backbone of B. Finally, one of the three samples identified as CRF06_cpx (CY422) clustered with the reference sequence for CRF32_06A6 with a 100% rather than clustering with the reference sequence for CF06_cpx. Bootscan and similarity plot analyses classified this sample as “Rec. of 32_06A6, G”. Ultimately, for the confirmation of their unique mosaic structures, preliminary bootscan, and similarity plot analyses were performed on each of the 24 ‘discrepant samples’ identified as an uncharacterized recombinant HIV-1 strain by REGA 3.0. In two different follow-up studies, 14 of the 24 samples were characterized as four novel CRFs, CRF91_cpx [25], CRF129_56G, CRF130_A1B, and CRF131_A1B (C. Topcu et al., manuscript in preparation for publication). A total of 9 of the 10 remaining samples were discovered to have unique mosaic structures that were not previously described, confirming that they were uncharacterized recombinant HIV-1 strains, while the remaining sample was identified as belonging to the CRF56_cpx lineage.

According to the phylogenetically based data derived from the HIV-1 near-full-length genome sequences, four of the 38 ‘discrepant samples’ were identified as belonging to a pure group M subtype (*n* = 4, 10.5%), eight were CRFs (*n* = 8, 21.1%) and 26 were uncharacterized recombinant HIV-1 strains (*n* = 26, 68.4%). In particular, all four samples belonging to a pure group M subtype were identified as sub-subtype A6 (*n* = 4, 10.5%). Among those eight samples belonging to previously characterized CRF lineages, four were CRF02_AG (*n* = 4, 10.5%), two were CRF06_cpx (*n* = 2, 5.3%), one was CRF04_cpx (*n* = 1, 2.6%), and one was CRF56_cpx (*n* = 1, 2.6%). Consequently, among the 26 uncharacterized recombinant HIV-1 strains, 14 were classified into four novel CRFs in two follow-up studies, and the remaining 12 were confirmed to have unique mosaic structures, which were not previously described and could be further classified into 12 novel URFs.

In retrospective analyses of the subtypes determined based on *pol* region sequences, it was discovered that all except one of the tested subtyping tools incorrectly identified the four samples belonging to pure group M sub-subtype A6 as sub-subtype A1. Stanford was the only HIV-1 subtyping tool to correctly determine the parental lineage as subtype A for all four samples without further classifying them into sub-subtypes. For the 34 HIV-1 recombinants belonging to CRFs and uncharacterized recombinant strains, using near-full-length genome sequences, REGA 3.0 correctly determined the HIV-1 genotypic subtypes of the 19 samples, which included seven CRFs and 12 uncharacterized recombinant HIV-1 strains. However, the rest of the subtyping tools showed significantly lower accuracy rates when determining the subtypes of recombinants.

## 4. Discussion

In this study, we developed a comprehensive near-full-length HIV-1 genome RT–PCR and sequencing assay in an effort to delineate the accuracy of phylogenies based on the analysis of HIV-1 genomes of increasing length. Using HIV-1 *PR/RT* (2253–3359 in the HXB2 genome) and *pol* region (2253–5250 in the HXB2 genome) sequences derived from 134 consenting newly diagnosed or chronically HIV-1-infected patients, we demonstrated a significant increase in the consistency of subtype classification among different subtyping tools when longer nucleotide sequences were analyzed. The results of the study also showed that, regardless of the decrease in discrepancies with longer sequence lengths, if a discrepancy persists in the subtyping results of the *pol* region, then there is a high likelihood that the query sequence belongs to a recombinant HIV-1 strain. Additionally, among the six different subtyping tools tested, REGA 3.0 [16], COMET 2.3 [17], jpHMM [18], SCUEAL [19], Stanford [20,21], and Geno2pheno 3.4 [22], REGA 3.0 was established as the best-performing tool for subtyping recombinant HIV-1 strains, while Stanford was shown to identify pure group M subtypes more accurately. However, the subtyping results of 38 near-full-length HIV-1 genome (790–8795 in the HXB2 genome) sequences illustrated that *pol* region sequences, which presented higher consistency compared to the *PR/RT* region, were not representative of the near-full-length HIV-1 genomes. Our study highlights that only near-full-length or full-length HIV-1 genome sequences provide sufficiently accurate results for determining HIV-1 genotypic subtypes, especially in polyphyletic HIV-1 epidemics characterized by high genetic diversity. Furthermore, this study draws attention to the increasing trend in the emergence of novel recombinant HIV-1 strains in regions with high HIV-1 genetic diversity. The results of this study highlight the significance of near-full-length or full-length HIV-1 genome sequencing to understand the extent of the complexity of HIV-1 genetic diversity and to evaluate the actual prevalence of uncharacterized HIV-1 recombinants for the identification of possible novel URFs and CRFs.

The study participants were recruited from the Grigorios HIV Clinic of Larnaca General Hospital, which is the single national clinical service for the care of HIV-1-infected patients in Cyprus. With a consent rate over 95%, this provides a unique opportunity for acquiring a cohort representative of the entire HIV-1 epidemic in Cyprus. Thus, with regard to the clinical, epidemiological, and behavioral information provided by the study patients, the HIV-1 epidemic in Cyprus was revealed to be characterized by young Cypriot MSM between the ages of 31 and 40 who reported being infected in Cyprus and residing in Nicosia. The preliminary phylogenetic- and phylodynamic-based results of a prospective molecular epidemiology study (C. Topcu et al., manuscript in preparation for publication) conducted from 2017 to 2021 support this finding. Most of the numerous HIV-1 transmission clusters identified in this prospective molecular epidemiology study (C. Topcu et al., manuscript in preparation for publication) were characterized by a similar cohort, demonstrating that the young Cypriot MSM community is, in fact, the main driving force of HIV-1 transmission in Cyprus. Although most of the patients originated from Cyprus, the remainder of the cohort (46.3%) was reported to have originated from 17 other countries within the borders of Africa, Europe, and Asia. A molecular epidemiology study with dense sampling that collected data from 1986 to 2012 in Cyprus illustrated that a significant proportion of the assembled cohort (23.5%) originated from Africa, Europe, and Asia [7]. The findings of this study show an increased rate of migration from these continents, resulting in an influx of various HIV-1 group M subtypes and CRFs into Cyprus.

It should be emphasized that previously conducted molecular epidemiology studies with dense sampling showed that the HIV-1 epidemic of Cyprus is highly polyphyletic, with an increasing trend of genetic diversity [7,8,23,26,27]. Additionally, the preliminary results of a prospective molecular epidemiology study (C. Topcu et al., manuscript in preparation for publication) conducted from 2017 to 2021 show that the polyphyletic HIV-1 epidemic persists in Cyprus. It is well established that the probability of the generation of new recombinant HIV-1 strains increases in polyphyletic HIV-1 epidemics due to the increased possibility of coinfection of individuals with two or more highly divergent HIV-1 strains [9]. However, due to the mosaic genomic structure of recombinant HIV-1 strains, HIV-1 genotypic subtypes differ based on the length of the nucleotide sequence and the region of the HIV-1 genome being analyzed. Furthermore, variation among the subtyping results of different subtyping tools has been previously reported in a study that compared subtyping tools based on *PR/RT* sequences [28]. Accordingly, we hypothesized that HIV-1 genotypic subtypes determined based on partial sequences will not accurately reveal the correct subtype of the entire HIV-1 genome, especially in polyphyletic HIV-1 epidemics with a high prevalence of uncharacterized recombinant HIV-1 strains.

As such, using the HIV-1 *PR/RT* (2253–3359 in the HXB2 genome) and *pol* region (2253–5250 in the HXB2 genome) sequences isolated from 134 study participants, we evaluated the subtyping results of both regions using six different subtyping tools in an effort to compare the derived phylogenies against near-full-length HIV-1 genome sequences. The accuracy of subtype interpretation by the best-performing subtyping tool was evaluated with regard to the gold standard comparative phylogenetic analysis method for all three regions. The total number of discrepancies generated in the *PR/RT* and *pol* regions were calculated to reveal the correlation between the length of the nucleotide sequence and the consistency of subtypes among different subtyping tools. Accordingly, this study showed that 39.6% of the 134 *PR/RT* region sequences showed disagreement in the subtyping results among the different subtyping tools, while only 28.4% of the 134 *pol* region sequences showed disagreement. The results showed an 11.2% decrease in the discrepancies generated by subtyping tools when longer nucleotide sequences were employed for subtyping. Therefore, HIV-1 *pol* region sequences were concluded to generate more consistent subtyping results and, hence, were adopted for the following comparative analyses. Similarly, in a previous study in which the performance of eight subtyping tools was evaluated, it was revealed that *PR* sequences presented lower performance and higher variation among the subtyping results of different tools compared to longer *RT* sequences [16]. Moreover, we evaluated the best-performing subtyping tool among the six different tools used in this study by investigating the number of discrepancies generated by each subtyping tool. The findings of this study indicated that REGA 3.0 generated the fewest discrepancies collectively in both regions and, hence, was considered the best-performing tool among the tested tools.

With respect to the obtained subtyping results of the 134 patient samples based on the *pol* region sequences determined by REGA 3.0, the polyphyletic HIV-1 epidemic in Cyprus was characterized by the highest prevalence of sub-subtype A1 (23.9%), followed by subtype B (23.1%). The third most predominant subtype was CRF02_AG (14.9%). The increased prevalence of CRF02_AG could be explained by the increased rate of migration from Western and Central Africa to Cyprus, as discovered in a prospective molecular epidemiology study (C. Topcu et al., manuscript in preparation for publication) [29]. Moreover, five other pure group M subtypes and five other CRF lineages with various predominance have been identified in the polyphyletic HIV-1 epidemic of Cyprus. More importantly, the results highlighted that 20.1% of the entire cohort harbored uncharacterized recombinant HIV-1 strains, demonstrating the elevated emergence of novel HIV-1 recombinants in HIV-1 epidemics characterized by high genetic diversity.

The comparative evaluation of the subtyping results of the 38 ‘discrepant samples’ that presented disagreement in both the *PR/RT* and *pol* regions or only in the *pol* region, against phylogenetic clustering confirmed the high accuracy of the HIV-1 genotypic subtypes determined by REGA 3.0. This could be observed in the phylogenetic tree, as the subtype classifications were mostly consistent and agreed with the HIV-1 clades. Additionally, REGA 3.0 was previously shown to perform better in subtyping CRFs than other subtyping tools, which is advantageous in the evaluation of polyphyletic HIV-1 epidemics characterized by a number of CRFs [16]. The subtyping results of the *pol* region sequences of the 38 ‘discrepant samples’ mostly identified the strains as uncharacterized recombinant HIV-1 strains (68.4%), suggesting that the HIV-1 genotypic subtypes of uncharacterized HIV-1 recombinants tend to show variation between different subtyping tools. Therefore, we proceeded with near-full-length HIV-1 genome sequencing of the 38 ‘discrepant samples’ with the purpose of identifying the most accurate HIV-1 phylogenies.

Henceforth, we developed a comprehensive near-full-length HIV-1 genome RT–PCR assay (790–8795 in the HXB2 genome) consisting of three overlapping amplicons spanning the whole genome amplified via a previously described HIV-1 pol RT–PCR assay (2253–5250 in the HXB2 genome) [13], HIV-1 gag RT–PCR assay (790–2292 in the HXB2 genome) and HIV-1 env RT–PCR assay (5041–8795 in the HXB2 genome). A total of 29 sequencing primers were designed to obtain the near-full-length HIV-1 genome sequences, providing high primer coverage at each nucleotide position. Twenty-three of the 29 sequencing primers showed over a 90% success rate, ranging from 90% to 100%, minimizing the possibility of failure in sequencing. Broad HIV-1 subtype coverage allowed the amplification and sequencing of the highly prevalent recombinant HIV-1 strains in our cohort. In addition, touchdown PCR was adopted for all three assays to increase the specificity of the assays by reducing nonspecific binding, which could potentially arise due to comprehensive primer design. Additionally, as previously reported, the HIV-1 pol RT–PCR assay showed a 97.9% success rate [13], while the HIV-1 gag and env RT–PCR assays exhibited 100% and 84.4% success rates, respectively. The lower success rate of the HIV-1 env RT–PCR assay was caused by the failure of HIV-1 env region sequencing in seven samples, which were excluded from this study to ensure the coherence of the data. The sequencing of this region failed due to low-quality reads, which potentially resulted from the high prevalence of quasispecies arising from mutations in this less conserved region of the HIV-1 genome.

The obtained near-full-length HIV-1 genome (790–8795 in the HXB2 genome) sequences of the 38 ‘discrepant samples’ were analyzed for HIV-1 genotypic subtypes using REGA 3.0. The following comparative phylogenetic analyses performed against a reference dataset of pure group M subtypes and CRFs (RIP Alignment 2017) confirmed that the subtyping results of REGA 3.0 were mostly consistent and agreed with the phylogenetic clustering results, demonstrating the high accuracy of subtype classification. More detailed analyses of the maximum likelihood tree accompanied by bootscan and similarity plot analyses revealed that most of the ‘discrepant samples’ were uncharacterized HIV-1 recombinants (68.4%), while the rest were CRFs (21.1%) and pure group M subtypes (10.5%). The study highlighted that 89.5% of the discrepancies were caused by recombinant HIV-1 strains, either characterized or uncharacterized, indicating that a discrepant subtyping result among the subtyping tools suggests that the query sequence belongs to a recombinant HIV-1 strain. It is noteworthy that the discrepancy rate increased significantly when the near-full-length genome sequences of the 38 ‘discrepant samples’ were analyzed in four of the six HIV-1 subtyping tools that can analyze full-length genome sequences (REGA 3.0, COMET 2.3, jpHMM and Stanford).

This phenomenon of discrepant subtyping results generated by different subtyping tools was previously exploited in a study for the selection of samples for the characterization of new recombinant HIV-1 strains as novel URFs [30]. In fact, in this study, REGA HIV-1 and 2 Automated Subtyping Tool Version 2.0 (REGA 2.0) [31] and, to a somewhat lesser degree, REGA 3.0 were discovered to perform better than other tools when giving an indication of a possible URF. Indeed, five of the 38 ‘discrepant samples’ (CY467, CY494, CY520, CY533, and CY614) in our cohort were recently identified and characterized as a novel CRF, CRF91_cpx, by our laboratory [25]. Additionally, nine samples (CY448, CY525, CY526, CY529, CY537, and CY625; CY413; CY584 and CY620) from this cohort were very recently discovered to belong to three additional novel CRFs and were denoted CRF129_56G, CRF130_A1B and CRF131_A1B by the Los Alamos HIV Sequence Database in accordance with the standards of the HIV nomenclature (https://www.hiv.lanl.gov/content/sequence/HelpDocs/subtypes-more.html, accessed on 12 September 2022) (C. Topcu et al., manuscript in preparation for publication).

All samples in this cohort that belonged to one of the four abovementioned novel CRFs generated at least one discrepant subtyping result among the six tested tools and were also identified as uncharacterized HIV-1 recombinants by REGA 3.0 based on *pol* region sequences. Thus, REGA 3.0 shows advantages over the five other tools tested in this study for the investigation of recombinant HIV-1 strains in polyphyletic epidemics comprising a high percentage of uncharacterized recombinant HIV-1 strains. Regardless, the results suggest that the subtyping of the *pol* region is not sufficiently accurate to define the correct phylogeny of the entire HIV-1 genome, especially if the analyzed sample exhibits discrepancies among different subtyping tools. Additionally, it was shown that even though REGA 3.0 presented the highest accuracy, correctly subtyping 19 of the 34 recombinant HIV-1 strains (55.9%) based on near-full-length HIV-1 genomes, further recombination analysis is essential for defining the correct phylogenies of recombinant strains. In contrast, the retrospective analyses of the HIV-1 genotypic subtypes determined based on *pol* region sequences revealed that Stanford presented the highest accuracy in determining subtypes of samples belonging to pure group M lineages.

## 5. Conclusions

The results demonstrated that the discrepancy among the different subtyping tools was significantly reduced from 39.6% to 28.4% when *pol* region sequences were used rather than *PR/RT* region sequences, indicating that the increase in nucleotide sequence length is directly correlated with more consistent subtyping results. The results also revealed that if the discrepancy persists in the subtyping results of the *pol* region, then there is a high likelihood, of 89.5%, that the query sequence is an HIV-1 recombinant, 68.4% of which belong to uncharacterized recombinant HIV-1 strains. REGA 3.0 was the best-performing HIV-1 subtyping tool among the tested tools, as it generated the lowest number of discrepancies. Although REGA 3.0 was demonstrated to present a better performance in the subtyping of recombinant HIV-1 strains, which is particularly advantageous in HIV-1 epidemics characterized by high genetic diversity and an elevated prevalence of uncharacterized HIV-1 strains, Stanford was observed to perform better in defining phylogenies of pure group M subtypes. While the comparative phylogenetic analyses mostly confirmed the accuracy of the HIV-1 genotypic subtypes determined by REGA 3.0 for both *PR/RT* and *pol* regions, it was observed that subtypes determined based on partial nucleotide sequences were not representative of the near-full-length HIV-1 genomes. It was concluded that, especially in populations with polyphyletic HIV-1 epidemics resulting in a high prevalence of complex recombinant strains, neither *PR/RT* nor *pol* region nucleotide sequences are reliable for the determination of HIV-1 genotypic subtypes, and only near-full-length or full-length HIV-1 genome sequences are sufficiently accurate. Thus, this study emphasizes the importance of near-full-length or full-length HIV-1 genome sequencing to obtain clearer insights into the genetic diversity of polyphyletic HIV-1 epidemics and to evaluate the actual prevalence of uncharacterized HIV-1 recombinants for the identification of possible novel URFs and CRFs.

## Figures and Tables

**Table 1:** PCR thermal cycling conditions of the HIV-1 pol RT–PCR assay.

**Primary Touchdown RT–PCR ** *^**a**^*			
**Step *^c^***	**Temperature (°C)**	**Time (min)**	**Number of Cycles**
Reverse Transcription	50	10:00	1×
RT Inactivation/Initial Denaturation	98	2:00	1×
Initial Amplification	98	0:10	10×
	61–52 (ΔΤ = −1 °C) *^d^*	0:10	
	72	2:00	
Final Amplification	98	0:10	30×
	52	0:10	
	72	2:00	
Final Extension	72	5:00	1×
Reaction Stop	4	Indefinitely	Hold
**Nested Touchdown PCR *^b^***			
**Step**	**Temperature (** **°C)**	**Time (min)**	**Number of Cycles**
Initial Denaturation/Tag Polymerase Activation	94	2:00	1×
Initial Amplification	94	0:30	10×
	62–53 (ΔΤ = −1 °C)	0:30	
	72	3:30	
Final Amplification	94	0:30	30×
	53	0:30	
	72	3:30	
Final Extension	72	5:00	1×
Reaction Stop	4	Indefinitely	Hold

*^a^* PCR thermal cycling conditions for cDNA synthesis through primary touchdown RT–PCR. *^b^* PCR thermal cycling conditions for amplification of the primary touchdown RT–PCR product through nested touchdown PCR. *^c^* ‘Step’ corresponds to each stage of the polymerase chain reaction. *^d^* Initial amplification consists of a 10 s annealing step that starts at 10 °C higher than the optimal primer melting temperature (Tm), with a ΔΤ = −1 °C in each subsequent cycle, until the optimal Tm is reached at the 10th cycle.

**Table 2:** PCR and sequencing primers for near-full-length HIV-1 genome sequencing.

Designation *^a^*	Target Gene	Sequence *^b^*	Position *^c^*	Amplicon Length (nts)	Reference
**PCR Primers**					
630 (F)	*gag*	TAGCAGTGGCGCCC	630–643	1872	This study
2501 (R)	*gag*	GTTGACAGGTGTAGGTCCTAC	2481–2501		[23]
684 * (F)	*gag*	TCTCGACGCAGGACTCG	684–700	1721	[23]
2404 * (R)	*gag*	CCAATTCCYCCTATCATTTTTGGTTTCC	2377–2404		This study
1810 (F)	*pol*	GCTACAYTAGAAGAAATGATGACAGCATG	1810–1838	3766	[13]
5575 * (R)	*pol*	TCTGGGGCTTGTTCCATCTATC	5554–5575		[13]
2006 (F)	*pol*	GGGCCCCTAGGAAAAAGGG	2006–2024	3259	[13]
5264 (R)	*pol*	CCTGTATGCAGACCCCAATATGTT	5241–5264		[13]
3777 * (F)	*env*	TGGATTCCTGARTGGGARTTTG	3777–3798	5405	[13]
9181 (R)	*env*	GTGTGTARTTYTGCCAATCAGG	9160–9181		This study
4155 * (F)	*env*	GTACCAGCACACAAAGGRATTG	4155–4176	4883	[13]
9038 (R)	*env*	TAAGTCATTGGTCTTARAGGYACYTG	9013–9038		This study
**Sequencing Primers**					
684 * (F)	*gag*	TCTCGACGCAGGACTCG	684–700		[23]
1173 (F)	*gag*	CAGYCAAAATTAYCCTATAGTGCA	1173–1196		[23]
1985 (R)	*gag*	CCTTCYTTGCCACARTTGAAACAY	1962–1985		[23]
2404 * (R)	*gag*	CCAATTCCYCCTATCATTTTTGGTTTCC	2377–2404		This study
2454 (F)	*pol*	GGAMAWAARGCTATAGGTACAG	2454–2475		[13]
2610 (R)	*pol*	CYTTTGGGCCATCCATTC	2593–2610		[13]
3003 (F)	*pol*	GGATGGAAAGGATCACC	3003–3019		[13]
3019 (R)	*pol*	GGTGATCCTTTCCATCC	3003–3019		[13]
3462 (R)	*pol*	CTGCCARTTCTARYTCTGCTTC	3441–3462		[13]
3777 * (F)	*pol*	TGGATTCCTGARTGGGARTTTG	3777–3798		[13]
4060 (R)	*pol*	CCTAATGCATAYTGTGAGTCTGTTAC	4035–4060		[13]
4155 * (F)	*pol*	GTACCAGCACACAAAGGRATTG	4155–4176		[13]
4324 (F)	*pol*	TAGCAAAAGAAATAGTAGCCAGCTG	4324–4348		[13]
4558 (R)	*pol*	ACTGGCCATCTTCCTGCTAATTTTA	4534–4558		[13]
4776 (F)	*env*	CACAATTTTAAAAGAAAAGGGGGGATTG	4776–4803		This study
5554 (F)	*env*	GATAGATGGAACAAGCCCCAGA	5554–5575		This study
5575 * (R)	*env*	TCTGGGGCTTGTTCCATCTATC	5554–5575		[13]
5960 (F)	*env*	GGCATHTCCTATGGCAGGAAG	5960–5980		This study
6203 (F)	*env*	GAAAGAGCAGAAGAYAGTGGMA	6203–6224		This study
6438 (F)	*env*	CATGCCTGTGTACCCACAGA	6438–6457		[23]
6457 (R)	*env*	TCTGTGGGTACACAGGCATG	6438–6457		This study
6858 (F)	*env*	CCAATTCCYATACATTATTGTGCYC	6858–6882		[23]
6882 (R)	*env*	GRGCACAATAATGTATRGGAATTGG	6858–6882		This study
7519 (F)	*env*	AAGCAATGTATGCCCCTCC	7519–7537		This study
7537 (R)	*env*	GGAGGGGCATACATTGCTT	7519–7537		This study
8039 (R)	*env*	GGTGCARATGWGTTTTCCAGAGC	8017–8039		[23]
8339 (F)	*env*	AATAGAGTTAGGCAGGGATACTCACC	8339–8364		This study
8365 (R)	*env*	GGTGAGTATCCCTGCCTAACTCTATT	8340–8365		This study
9011 (R)	*env*	GGYCTGACTGGAAARCCYAC	8992–9011		This study

*^a^* Reverse transcriptase, primary and secondary PCR primers, and sequencing primers named as it appears in the text; Orientation of the PCR primer is indicated in parenthesis: F, forward; R, reverse. *^b^* Y indicates an equal molar mixture of C and T; R, A, and G; M, A, and C; W, A, and T; H, A, C, and T. *^c^* Primer positions correspond to subtype B HIV-1 HXB2 strain (Genbank accession number K03455). * Primers, which are used both as PCR and sequencing primers.

**Table 3:** PCR thermal cycling conditions of the HIV-1 gag RT–PCR assay.

**Primary Touchdown RT–PCR ** *^**a**^*			
**Step *^c^***	**Temperature (°C)**	**Time (min)**	**Number of Cycles**
Reverse Transcription	50	10:00	1×
RT Inactivation/Initial Denaturation	98	2:00	1×
Initial Amplification	98	0:10	10×
	64–55 (ΔΤ = −1 °C) *^d^*	0:10	
	72	1:00	
Final Amplification	98	0:10	15×
	55	0:10	
	72	1:00	
Final Extension	72	5:00	1×
Reaction Stop	4	Indefinitely	Hold
**Nested Touchdown PCR *^b^***			
**Step**	**Temperature (** **°C)**	**Time (min)**	**Number of Cycles**
Initial Denaturation/Tag Polymerase Activation	94	2:00	1×
Initial Amplification	94	0:30	10×
	64–55 (ΔΤ = −1 °C)	0:30	
	72	1:00	
Final Amplification	94	0:30	30×
	55	0:30	
	72	1:00	
Final Extension	72	5:00	1×
Reaction Stop	4	Indefinitely	Hold

*^a^* PCR thermal cycling conditions for cDNA synthesis through primary touchdown RT–PCR. *^b^* PCR thermal cycling conditions for amplification of the primary touchdown RT–PCR product through nested touchdown PCR. *^c^* ‘Step’ corresponds to each stage of the polymerase chain reaction. *^d^* Initial amplification consists of a 10 s annealing step that starts at 10 °C higher than the optimal primer melting temperature (Tm), with a ΔΤ = −1 °C in each subsequent cycle, until the optimal Tm is reached at the 10th cycle.

**Table 4:** PCR thermal cycling conditions of the HIV-1 env RT–PCR assay.

**Primary Touchdown RT–PCR *^a^***			
**Step *^c^***	**Temperature (°C)**	**Time (min)**	**Number of Cycles**
Reverse Transcription	58	10:00	1×
RT Inactivation/Initial Denaturation	98	2:00	1×
Initial Amplification	98	0:10	10×
	69–60 (ΔΤ = −1 °C) *^d^*	0:10	
	72	3:00	
Final Amplification	98	0:10	30×
	60	0:10	
	72	3:00	
Final Extension	72	5:00	1×
Reaction Stop	4	Indefinitely	Hold
**Nested PCR *^b^***			
**Step**	**Temperature (** **°C)**	**Time (min)**	**Number of Cycles**
Initial Denaturation/Tag Polymerase Activation	98	0:30	1×
Amplification	98	0:10	40×
	60	0:10	
	72	3:00	
Final Extension	72	5:00	1×
Reaction Stop	4	Indefinitely	Hold

*^a^* PCR thermal cycling conditions for cDNA synthesis through primary touchdown RT–PCR. *^b^* PCR thermal cycling conditions for amplification of the primary touchdown RT–PCR product through nested PCR. *^c^* ‘Step’ corresponds to each stage of the polymerase chain reaction. *^d^* Initial amplification consists of a 10 s annealing step that starts at 10 °C higher than the optimal primer melting temperature (Tm), with a ΔΤ = −1 °C in each subsequent cycle, until the optimal Tm is reached at the 10th cycle.

**Table 5:** Comparison of subtyping results of 38 ‘discrepant samples’ based on ‘*PR/RT’* and ‘*POL’* region nucleotide sequences utilizing six subtyping tools.

No.	Samples ^1^	GenBank Acession Number	REGA 3.0 ^4^		COMET 2.3 ^5^		jpHMM ^6^		SCUEAL ^7^		Stanford ^8^		Geno2pheno 3.4 ^9^		Discrepancy ^10^		
			PR/RT ^2^	POL ^3^	PR/RT	POL	PR/RT	POL	PR/RT	POL	PR/RT	POL	PR/RT	POL	PR/RT	POL	Regions
**3**	CY394	ON989215	CRF 35_AD	Rec. of 35_AD, G	35_AD	Unassigned; 35_AD, 14_BG, G, 20_BG, G, 24_BG, G, 14_BG, G	A1 D	A1 D G	A1, D recombinant	Complex	CRF35_AD	A + D	A1	A1	YES	YES	YES
**4**	CY395	ON989216	Rec. of 14_BG, A1	Rec. of A1, G	Unassigned; G, A1	Unassigned; A1, G	A1 G	A1 G	A1, G recombinant	A1, G recombinant	G	A	14_BG	A1	YES	YES	YES
**7**	CY399	ON989219	G-like	Rec. of 02_AG, G, A1	02_AG	02_AG	G	A1 G	G	Complex	CRF02_AG	CRF02_AG	02_AG	02_AG	YES	YES	YES
**8**	CY401	ON989220	A1	A1	A1	A1	A1	A1	A1	A1, G recombinant	A	A	01_AE	A1	YES	YES	YES
**14**	CY413	ON989226	B	Rec. of B, A1	Unassigned; B, A1	Unassigned; B, A1	B	A1 B	B, G recombinant	A1, B recombinant	B	B	B	B	YES	YES	YES
**18**	CY422	ON989230	CRF06_CPX	CRF06_CPX	06_cpx	06_cpx	D	A1 C D G J	CRF32-like	Complex	CRF06_cpx	CRF06_cpx	06_CPX	06_CPX	YES	YES	YES
**27**	CY448	ON989239	Rec. of B, G	Rec. of B, A1, G	Unassigned; 02_AG, 56_cpx	Unassigned; 56_cpx, 02_AG, 63_02A1, 02_AG, 71_BF1, 25_cpx, 02_AG, C, 43_02G, 02_AG, 71_BF1, 05_DF, 71_BF1, 05_DF, 02_AG, A1, B	B G	A1 B G	B, G recombinant	Complex	B + CRF02_AG	B + CRF02_AG	02_AG	B	YES	YES	YES
**33**	CY457	ON989245	B	Rec. of B, A1	B	Unassigned; B, A1	B	A1 B	B	A3, B recombinant	B	B	B	B	-	YES	YES
**36**	CY462	ON989248	CRF 06_CPX	CRF 06_CPX	06_cpx	06_cpx	A1 C G	A1 C G J	G, K recombinant	CRF06-like	CRF06_cpx	CRF06_cpx	06_CPX	06_CPX	YES	YES	YES
**40**	CY467	OK584018	Rec. of G, A1	Rec. of G, A1, B	02_AG	Unassigned; 02_AG, 11_cpx, 01_AE, 90_BF1, B, D, B, D, B, D, B	G	A1 B G	G	Complex	CRF02_AG	CRF02_AG	02_AG	02_AG	YES	YES	YES
**42**	CY471	ON989253	A1	Rec. of A1, B	A1	Unassigned; A1, B	A1	A1 B	A1	A1, B recombinant	A	A	A1	A1	-	YES	YES
**44**	CY473	ON989255	B	Rec. of B, G, A1	B	Unassigned; 02_AG, 38_BF1, B, 94_cpx, 08_BC, B, 08_BC, B, 07_BC, 90_BF1, 07_BC, 52_01B, 07_BC, 90_BF1, B, 68_01B, B, 90_BF1, B	B G	A1 B G	Complex	Complex	B	B	B	B	YES	YES	YES
**54**	CY488	ON989265	Rec. of B, A1	Rec. of B, A1	Unassigned; 56_cpx, 02_AG, 90_BFI, 72_BFI, 90_BFI, 83_cpx	Unassigned; 56_cpx, 02_AG, 90_BF1, 72_BF1, 90_BF1, 83_cpx, 90_BF1, A1, 02_AG, 19_cpx, B, 90_BF1	A1 B	A1 B	Complex	Complex	B + CRF02_AG	B + CRF02_AG	02_AG	03_AB	YES	YES	YES
**58**	CY494	OK283056	Rec. of G, A1	Rec. of 02_AG, G	02_AG	Unassigned; 02_AG, 11_cpx, 01_AE	G	A1 G	G	A-ancestral, G recombinant	CRF02_AG	CRF02_AG	02_AG	02_AG	YES	YES	YES
**65**	CY508	ON989275	G	Rec. of G, K	G	Unassigned; G, A1, D	G	B G	G	G, J recombinant	G	G	G	G	-	YES	YES
**68**	CY515	ON989278	Rec. of F1, B	Rec. of B, F1	F1	Unassigned; B, F1, A1, F1, F2	B F1	B F1	Complex	B, F1 recombinant	B	B	12_BF	B	YES	YES	YES
**70**	CY519	ON989280	F1	Rec. of F1, A1	Unassigned; 71_BF1, A1	Unassigned; F1, A1, 01_AE, A1, G	F1	A1 F1	F1	Complex	F	F	F1	F2	YES	YES	YES
**71**	CY520	OK283057	Rec. of G, A1	Rec. of 02_AG, G	02_AG	Unassigned; 02_AG, 11_cpx, 01_AE	G	A1 G	G	Complex	CRF02_AG	CRF02_AG	02_AG	02_AG	YES	YES	YES
**73**	CY525	ON989282	Rec. of G, B	Rec. of B, A1, G	Unassigned; 02_AG, 56_cpx	Unassigned; 02_AG, 56_cpx, 90_BF1, B, 90_BF1, B, 56_cpx, B, 56_cpx, B, 39_BF, 51_01B, B, 51_01B, B, 90_BF1, 56_cpx, 90_BF1	B C G	A1 B G	Complex	Complex	B + CRF02_AG	B + CRF02_AG	02_AG	B	YES	YES	YES
**74**	CY526	ON989283	Rec. of G, B	Rec. of B, A1, G	Unassigned; 02_AG, 56_cpx	Unassigned; 02_AG, 56_cpx, 90_BF1, B, 90_BF1, B, 56_cpx, B, 39_BF, 90_BF1, 39_BF, B, 69_01B, 90_BF1, 56_cpx	B G	A1 B G	Complex	Complex	B + CRF02_AG	B + CRF02_AG	02_AG	B	YES	YES	YES
76	CY529	ON989285	Rec. of G, B	Rec. of B, A1, G	Unassigned; 02_AG, 56_cpx	Unassigned; 02_AG, 56_cpx, 90_BF1, B, 90_BF1, B, 56_cpx, B, 56_cpx, B, 39_BF, 90_BF1, B, 90_BF1, 56_cpx	B C G	A1 B G	Complex	Complex	B + CRF02_AG	B + CRF02_AG	02_AG	B	YES	YES	YES
78	CY533	OK283058	Rec. of G, A1	Rec. of 02_AG, G, A1	02_AG	Unassigned; 02_AG, 11_cpx, 01_AE	G	A1 G	G	A, G recombinant	CRF02_AG	CRF02_AG	02_AG	02_AG	YES	YES	YES
80	CY535	ON989288	CRF 02_AG	CRF 02_AG	02_AG	02_AG	A1 D G	A1 C G	Complex	Complex	CRF02_AG	CRF02_AG	02_AG	02_AG	YES	YES	YES
81	CY537	ON989289	Rec. of G, B	Rec. of B, A1, G	Unassigned; 02_AG, 90_BF1, 31_BC, 56_cpx	Unassigned; 02_AG, 90_BF1, 31_BC, 56_cpx, 90_BF1, B, 90_BF1, B, 56_cpx, B, 39_BF, 90_BF1, 39_BF, B, 69_01B, 90_BF1, 56_cpx	B C G	A1 B G	Complex	Complex	B + CRF02_AG	B + CRF02_AG	02_AG	B	YES	YES	YES
82	CY538	ON989290	02_AG	Rec. of 02_AG, A1	02_AG	02_AG	G	A1 G	G	Complex	CRF02_AG	CRF02_AG	02_AG	02_AG	YES	YES	YES
84	CY541	ON989292	CRF 04_CPX	CRF 04_CPX	F1 (check for 04_cpx)	04_cpx	A1 C	A1 C	Complex	Complex	CRF04_cpx	CRF04_cpx	04_CPX	04_CPX	YES	YES	YES
85	CY542	ON989293	01_AE	01_AE	01_AE	01_AE	01_AE	01_AE	AE, B recombinant	AE, D recombinant	CRF01_AE	CRF01_AE	01_AE	01_AE	YES	YES	YES
86	CY543	ON989294	A1	A1	A1	A1	A1	A1	A1	A1	A	A	01_AE	01_AE	YES	YES	YES
95	CY555	ON989303	A1	A1	A1	A1	A1	A1	A1	A1	A	A	01_AE	01_AE	YES	YES	YES
107	CY576	ON989315	CRF 02_AG	CRF 02_AG	02_AG	02_AG	A1 G	A1 B G	Complex	Complex	CRF02_AG	CRF02_AG	02_AG	02_AG	-	YES	YES
110	CY581	ON989318	CRF 02_AG	Rec. of 02_AG, G, A1	02_AG	02_AG	A1 G	A1 G	G	A, G recombinant	CRF02_AG	CRF02_AG	02_AG	02_AG	YES	YES	YES
112	CY584	ON989320	B	Rec. of B, A1	B	Unassigned; B, A1	B	A1 B	B, D recombinant	A1, B recombinant	B	B	B	B	YES	YES	YES
125	CY611	ON989333	Rec. of 18_cpx, G	CRF 18_cpx	F1 (check for 18_cpx)	18_cpx	B G	A1 C F1 G	AE, G recombinant	G, K recombinant	CRF18_cpx	CRF18_cpx	K	02_AG	YES	YES	YES
126	CY612	ON989334	CRF 06_CPX	CRF 06_CPX	06_cpx	06_cpx	C G	A1 C G J	CRF06-like	Complex	CRF06_cpx	CRF06_cpx	06_CPX	06_CPX	YES	YES	YES
127	CY614	OK283059	Rec. of G, A1	Rec. of 02_AG, G, A1	02_AG	Unassigned; 02_AG, 11_cpx, 01_AE	G	A1 G	G	Complex	CRF02_AG	CRF02_AG	02_AG	02_AG	YES	YES	YES
128	CY615	ON989335	A1	A1	A1	A1	A1	A1	A1	A1	A	A	01_AE	01_AE	YES	YES	YES
132	CY620	ON989339	B	Rec. of B, A1	B	Unassigned; B, A1	B	A1 B	B, D recombinant	Complex	B	B	B	B	YES	YES	YES
134	CY625	ON989341	Rec. of B, G	Rec. of B, A1, G	Unassigned; 02_AG, B, 56_cpx	Unassigned; 02_AG, B, 56_cpx, 90_BF1, B, 56_cpx, B, 51_01B, B, 69_01B, 90_BF1	A1 B G	A1 B G	Complex	Complex	B + CRF02_AG	B + CRF02_AG	02_AG	B	YES	YES	YES

The cells highlighted in light-pink color show the discrepant subtyping of *‘PR/RT’* regions (corresponding to nucleotides 2253 to 3359 on the HXB2 genome), and the cells highlighted in light-gray color show the discrepant subtyping of ‘*POL*’ regions (corresponding to nucleotides 2253 to 5250 on HXB2 genome). ^1^ ‘Samples’ corresponds to the coded naming of study subject samples that contains five characters; e.g., in CY001, the first two characters, CY, denote Cyprus, the country of origin. The next three characters, 001, denote the study subject’s number. ^2^ ‘*PR/RT’* denotes nucleotide sequences of *protease* and partial *reverse transcriptase* genes (corresponding to nucleotides 2253 to 3359 on the HXB2 genome). ^3^ ‘*POL*’ denotes nucleotide sequences of *protease, reverse transcriptase, integrase,* and partial *vif* genes (corresponding to nucleotides 2253 to 5250 on the HXB2 genome). ^4^ REGA HIV-1 subtyping tool version 3.0 identifies the subtypes via using phylogenetic methods; the various recombinations are analyzed with bootscanning methods. It is available at: http://dbpartners.stanford.edu:8080/RegaSubtyping/stanford-hiv/typingtool/, accessed on 12 September 2022. ^5^ COMET 2.3 is the abbreviation for “COntext-based Modeling for Expeditious Typing”, while version 2.3 has additional support for HIV-1 nucleotide sequences for CRFs as well as URFs. It is available at: https://comet.lih.lu/, accessed on 12 September 2022. ^6^ jpHMM (‘jumping profile hidden Markov model’) predicts the genomic recombination of HIV-1 and the accurate breakpoints between the subtypes. It is available online at: http://jphmm.gobics.de/, accessed on 12 September 2022. ^7^ SCUEAL is an algorithm that can subtype only *pol* sequences. It is available at: http://classic.datamonkey.org/dataupload_scueal.php, accessed on 12 September 2022. ^8^ Stanford University curates the HIV Drug Resistance Database, which provides the public with the HIVdb Program. Its main function is to accept *pol* sequences and, in return, predict the resistance to various drugs, subtyping the sequence in parallel. It is available at: https://hivdb.stanford.edu/hivdb/by-sequences/, accessed on 12 September 2022. ^9^ Geno2pheno (resistance) 3.4 aligns HIV-1 *pol* gene nucleotide sequences to the HXB2 genome in order to predict resistance to ART drugs. It is available at: https://www.geno2pheno.org/index.php, accessed on 12 September 2022. ^10^ ‘Discrepancy’ denotes the disagreements in subtyping generated by the aforementioned subtyping tools. Discrepancy in the ‘*PR/RT’* region indicates the disagreements in the subtyping of each *PR/RT* nucleotide sequence (corresponding to nucleotides 2253 to 3359 on the HXB2 genome) among different subtyping tools. Discrepancy in the ‘*POL*’ region indicates the disagreements in the subtyping of each *pol* nucleotide sequence (corresponding to nucleotides 2253 to 5250 on the HXB2 genome) among different subtyping tools. Discrepancy in ‘Regions’ denotes the verdict of discrepant result, where the disagreements in both ‘*PR/RT’* and *‘POL’* regions or ‘*POL*’ region only indicate that there are discrepancies among the subtyping tools. Samples identified as discrepant in ‘Regions’ were selected to proceed with near-full-length HIV-1 genome sequencing.

**Table 6:** Genotypic subtyping results of 38 ‘discrepant samples’ based on near-full-length HIV-1 genome (NFLG) nucleotide sequences determined by the REGA HIV-1 subtyping tool, version 3.0.

No.	Samples ^1^	GenBank Acession Number	NFLG ^2^
1	CY394	ON989215	Rec. of 14_BG, A1, D
2	CY395	ON989216	Rec. of A1, G
3	CY399	ON989219	Rec. of G, 43_02G, A1
4	CY401	ON989220	A1
5	CY413	ON989226	Rec. of A1, B
6	CY422	ON989230	CRF06_CPX
7	CY448	ON989239	Rec. of 02_AG, B, G, A1
8	CY457	ON989245	B
9	CY462	ON989248	CRF06_CPX
10	CY467	OK584018	Rec. of 02_AG, G, B, J, D
11	CY471	ON989253	A1
12	CY473	ON989255	Rec. of 02_AG, B
13	CY488	ON989265	Rec. of 02_AG, B, G
14	CY494	OK283056	Rec. of 02_AG, G, J
15	CY508	ON989275	Rec. of G, 06_CPX
16	CY515	ON989278	Rec. of B, F1
17	CY519	ON989280	Rec. of F1, A1
18	CY520	OK283057	Rec. of 02_AG, G, J
19	CY525	ON989282	Rec. of 02_AG, B, G
20	CY526	ON989283	Rec. of 02_AG, B, G
21	CY529	ON989285	Rec. of 02_AG, B, G
22	CY533	OK283058	Rec. of 02_AG, G, J, A1
23	CY535	ON989288	CRF02_AG
24	CY537	ON989289	Rec. of 02_AG, B, G
25	CY538	ON989290	CRF02_AG
26	CY541	ON989292	CRF04_CPX
27	CY542	ON989293	Rec. of 01_AE, B
28	CY543	ON989294	A1
29	CY555	ON989303	A1
30	CY576	ON989315	CRF02_AG
31	CY581	ON989318	CRF02_AG
32	CY584	ON989320	Rec. of A1, B
33	CY611	ON989333	Rec. of 02_AG, F1, A1, G
34	CY612	ON989334	CRF06_CPX
35	CY614	OK283059	Rec. of 02_AG, G, J, A1
36	CY615	ON989335	A1
37	CY620	ON989339	Rec. of A1, B
38	CY625	ON989341	Rec. of 02_AG, B, G, A1

^1^ ‘Samples’ corresponds to the coded naming of study subject samples that contain five characters; e.g., in CY001, the first two characters, CY, denote Cyprus, the country of origin. The next three characters, 001, denote the study subject’s number. ^2^ ‘NFLG’ denotes nucleotide sequences of *gag, pol, vif, vpr, tat, rev, vpu,* and *env* genes (corresponding to nucleotides 790 to 8795 on HXB2 genome).

## Data Availability

The DNA nucleotide sequence data presented in this study are openly available in GenBank at https://www.ncbi.nlm.nih.gov/genbank/, accessed on 12 September 2022, and the accession numbers are ON989213-ON989341, OK584018, and OK283056-OK283059.

## Supplementary Materials

The following supporting information can be downloaded at https://www.mdpi.com/article/10.3390/v14102286/s1, Table S1: Comparison of subtyping results of 134 samples based on *‘PR/RT’* and *‘POL’* region nucleotide sequences utilizing six subtyping tools.

## References

[1] UNAIDS Global HIV & AIDS Statistics—Fact Sheet. https://www.unaids.org/sites/default/files/media_asset/UNAIDS_FactSheet_en.pdf.

[2] Lee C.-Y., Lin Y.-P., Wang S.-F., Lu P.-L. (2022). Late cART Initiation Consistently Driven by Late HIV Presentation: A Multicenter Retrospective Cohort Study in Taiwan from 2009 to 2019. Infect. Dis. Ther..

[3] Yang X., Su B., Zhang X., Liu Y., Wu H., Zhang T. (2020). Incomplete immune reconstitution in HIV/AIDS patients on antiretroviral therapy: Challenges of immunological non-responders. J. Leukoc. Biol..

[4] Hemelaar J., Elangovan R., Yun J., Dickson-Tetteh L., Kirtley S., Gouws-Williams E., Ghys P.D. (2020). Global and regional epidemiology of HIV-1 recombinants in 1990–2015: A systematic review and global survey. Lancet. HIV.

[5] Robertson D.L., Anderson J.P., Bradac J.A., Carr J.K., Foley B., Funkhouser R.K., Gao F., Hahn B.H., Kalish M.L., Kuiken C. (2000). HIV-1 nomenclature proposal. Science.

[6] Yamaguchi J., McArthur C., Vallari A., Sthreshley L., Cloherty G.A., Berg M.G., Rodgers M.A. (2019). Complete genome sequence of CG-0018a-01 establishes HIV-1 subtype L. J. Acquir. Immune Defic. Syndr..

[7] Pineda-Peña A.-C., Theys K., Stylianou D.C., Demetriades I., Abecasis A.B., Kostrikis L.G. (2018). HIV-1 Infection in Cyprus, the Eastern Mediterranean European Frontier: A Densely Sampled Transmission Dynamics Analysis from 1986 to 2012. Sci. Rep..

[8] Kostrikis L.G., Hezka J., Stylianou D.C., Kostaki E., Andreou M., Kousiappa I., Paraskevis D., Demetriades I. (2018). HIV-1 transmission networks across Cyprus (2010–2012). PLoS ONE.

[9] Robertson D.L., Hahn B.H., Sharp P.M. (1995). Recombination in AIDS viruses. J. Mol. Evol..

[10] Los Alamos HIV Sequence Database HIV Circulating Recombinant Forms (CRFs). https://www.hiv.lanl.gov/content/sequence/HIV/CRFs/crfs.comp.

[11] The EuroGUidelines Group for HIV Resistance (2001). EuroGuidelines Group for HIV Resistance Clinical and laboratory guidelines for the use of HIV-1 drug resistance testing as part of treatment management: Recommendations for the European setting. AIDS.

[12] World Health Organization Update of Recommendations on First- and Second-Line Antiretroviral Regimens. https://apps.who.int/iris/bitstream/handle/10665/325892/WHO-CDS-HIV-19.15-eng.pdf?ua=1.

[13] Chrysostomou A.C., Topcu C., Stylianou D.C., Hezka J., Kostrikis L.G. (2020). Development of a new comprehensive HIV-1 genotypic drug resistance assay for all commercially available reverse transcriptase, protease and integrase inhibitors in patients infected with group M HIV-1 strains. Infect. Genet. Evol..

[14] Kumar S., Stecher G., Li M., Knyaz C., Tamura K. (2018). MEGA X: Molecular Evolutionary Genetics Analysis across Computing Platforms. Mol. Biol. Evol..

[15] Larsson A. (2014). AliView: A fast and lightweight alignment viewer and editor for large datasets. Bioinformatics.

[16] Pineda-Peña A.-C., Faria N.R., Imbrechts S., Libin P., Abecasis A.B., Deforche K., Gómez-López A., Camacho R.J., de Oliveira T., Vandamme A.-M. (2013). Automated subtyping of HIV-1 genetic sequences for clinical and surveillance purposes: Performance evaluation of the new REGA version 3 and seven other tools. Infect. Genet. Evol..

[17] Struck D., Lawyer G., Ternes A.-M., Schmit J.-C., Bercoff D.P. (2014). COMET: Adaptive context-based modeling for ultrafast HIV-1 subtype identification. Nucleic Acids Res..

[18] Schultz A.-K., Zhang M., Bulla I., Leitner T., Korber B., Morgenstern B., Stanke M. (2009). jpHMM: Improving the reliability of recombination prediction in HIV-1. Nucleic Acids Res..

[19] Kosakovsky Pond S.L., Posada D., Stawiski E., Chappey C., Poon A.F.Y., Hughes G., Fearnhill E., Gravenor M.B., Leigh Brown A.J., Frost S.D.W. (2009). An evolutionary model-based algorithm for accurate phylogenetic breakpoint mapping and subtype prediction in HIV-1. PLoS Comput. Biol..

[20] Tang M.W., Liu T.F., Shafer R.W. (2012). The HIVdb System for HIV-1 Genotypic Resistance Interpretation. Intervirology.

[21] Liu T.F., Shafer R.W. (2006). Web resources for HIV type 1 genotypic-resistance test interpretation. Clin. Infect. Dis. Off. Publ. Infect. Dis. Soc. Am..

[22] Beerenwinkel N., Däumer M., Oette M., Korn K., Hoffmann D., Kaiser R., Lengauer T., Selbig J., Walter H. (2003). Geno2pheno: Estimating phenotypic drug resistance from HIV-1 genotypes. Nucleic Acids Res..

[23] Kousiappa I., van de Vijver D.A.M.C., Demetriades I., Kostrikis L.G. (2009). Genetic Analysis of HIV Type 1 Strains from Newly Infected Untreated Patients in Cyprus: High Genetic Diversity and Low Prevalence of Drug Resistance. AIDS Res. Hum. Retrovir..

[24] Thomson M.M., Casado G., Posada D., Sierra M., Nájera R. (2005). Identification of a novel HIV-1 complex circulating recombinant form (CRF18_cpx) of Central African origin in Cuba. AIDS.

[25] Topcu C., Georgiou V., Hezka Rodosthenous J., Demetriades I., Foley B.T., Kostrikis L.G. (2022). Characterization of a novel HIV-1 circulating recombinant form, CRF91_cpx, comprising CRF02_AG, G, J, and U, mostly among men who have sex with men. Virulence.

[26] Kousiappa I., Van De Vijver D.A.M.C., Kostrikis L.G. (2009). Near Full-Length Genetic Analysis of HIV Sequences Derived from Cyprus: Evidence of a Highly Polyphyletic and Evolving Infection. AIDS Res. Hum. Retrovir..

[27] Kousiappa I., Achilleos C., Hezka J., Lazarou Y., Othonos K., Demetriades I., Kostrikis L.G. (2011). Molecular characterization of HIV type 1 strains from newly diagnosed patients in Cyprus (2007–2009) recovers multiple clades including unique recombinant strains and lack of transmitted drug resistance. AIDS Res. Hum. Retrovir..

[28] Fabeni L., Berno G., Fokam J., Bertoli A., Alteri C., Gori C., Forbici F., Takou D., Vergori A., Zaccarelli M. (2017). Comparative Evaluation of Subtyping Tools for Surveillance of Newly Emerging HIV-1 Strains. J. Clin. Microbiol..

[29] Giovanetti M., Ciccozzi M., Parolin C., Borsetti A. (2020). Molecular Epidemiology of HIV-1 in African Countries: A Comprehensive Overview. Pathogens.

[30] Lunar M.M., Mlakar J., Zorec T.M., Poljak M. (2020). HIV-1 Unique Recombinant Forms Identified in Slovenia and Their Characterization by Near Full-Length Genome Sequencing. Viruses.

[31] de Oliveira T., Deforche K., Cassol S., Salminen M., Paraskevis D., Seebregts C., Snoeck J., van Rensburg E.J., Wensing A.M.J., van de Vijver D.A. (2005). An automated genotyping system for analysis of HIV-1 and other microbial sequences. Bioinformatics.

