# Supplementary material for: Comparative HIV-1 Phylogenies Characterized by PR/RT, Pol and Near-Full-Length Genome Sequences
Source: Viruses. 2022 Oct 17;14(10):2286. doi: 10.3390/v14102286 (PMC9608988; doi:10.3390/v14102286)
Supplement: Supplementary file 1 [file viruses-14-02286-s001.zip › Table S1.pdf]

Table S1. Comparison of subtyping results of 134 samples based on '*PR/RT*' and '*POL*' region nucleotide sequences utilizing six subtyping tools.

No.	Samples[1]	GenBank Accession Numbers	REGA 3.0[4]		COMET 2.3[5]		jpHMM[6]		SCUEAL[7]		Stanford[8]		Geno2pheno 3.4[9]		Discrepancy[10]		
			PR/RT[2]	POL[3]	PR/RT	POL	PR/RT	POL	PR/RT	POL	PR/RT	POL	PR/RT	POL	PR/RT	POL	Regions
1	CY391	ON989213	A1	A1	A1	A1	A1	A1	A1	A1	A	A	A1	A1	-	-	-
2	CY393	ON989214	CRF02_AG	CRF02_AG	02_AG	02_AG	A1 G	A1 G	Complex	Complex	CRF02_AG	CRF02_AG	02_AG	02_AG	-	-	-
3	CY394	ON989215	CRF 35_AD	Rec. of 35_AD, G	35_AD	Unassigned; 35_AD, 14_BG, G, 20_BG, G, 24_BG, G, 14_BG, G	A1 D	A1 D G	A1, D recombinant	Complex	CRF35_AD	A + D	A1	A1	YES	YES	YES
4	CY395	ON989216	Rec. of 14_BG, A1	Rec. of A1, G	Unassigned; G, A1	Unassigned; A1, G	A1 G	A1 G	A1, G recombinant	A1, G recombinant	G	A	14_BG	A1	YES	YES	YES
5	CY396	ON989217	CRF02_AG	CRF02_AG	02_AG	02_AG	G	A1 G	Complex	CRF02-like	CRF02_AG	CRF02_AG	02_AG	02_AG	YES	-	-
6	CY398	ON989218	B	B	B	B	B	B	B	B	B	B	B	B	-	-	-
7	CY399	ON989219	G-like	Rec. of 02_AG, G, A1	02_AG	02_AG	G	A1 G	G	Complex	CRF02_AG	CRF02_AG	02_AG	02_AG	YES	YES	YES
8	CY401	ON989220	A1	A1	A1	A1	A1	A1	A1	A1, G recombinant	A	A	01_AE	A1	YES	YES	YES
9	CY403	ON989221	B	B	B	B	B	B	B	B	B	B	B	B	-	-	-
10	CY405	ON989222	CRF02_AG	CRF02_AG	02_AG	02_AG	G	A1 G	Complex	Complex	CRF02_AG	CRF02_AG	02_AG	02_AG	YES	-	-
11	CY408	ON989223	A1	A1	A1	A1	A1	A1	A1	A1	A	A	A1	A1	-	-	-
12	CY409	ON989224	B	B	B	B	B	B	B	B	B	B	B	B	-	-	-
13	CY411	ON989225	B	B	B	B	B	B	B	B	B	B	B	B	-	-	-
14	CY413	ON989226	B	Rec. of B, A1	Unassigned; B, A1	Unassigned; B, A1	B	A1 B	B, G recombinant	A1, B recombinant	B	B	B	B	YES	YES	YES
15	CY414	ON989227	A1	A1	A1	A1	A1	A1	A1	A1	A	A	A1	A1	-	-	-
16	CY418	ON989228	B	B	B	B	B	B	B	B	B	B	B	B	-	-	-
17	CY421	ON989229	B	B	B	B	B	B	B	B	B	B	B	B	-	-	-
18	CY422	ON989230	CRF06_CPX	CRF06_CPX	06_cpx	06_cpx	D	A1 C D G J	CRF32-like	Complex	CRF06_cpx	CRF06_cpx	06_CPX	06_CPX	YES	YES	YES
19	CY423	ON989231	CRF02_AG	CRF02_AG	02_AG	A1 (check for 02_AG)	A1 G	A1 G	Complex	CRF02-like	CRF02_AG	CRF02_AG	02_AG	02_AG	-	-	-
20	CY424	ON989232	A1	A1	A1	A1	A1	A1	A1	A1	A	A	A1	A1	-	-	-
21	CY425	ON989233	B	B	B	B	B	B	B	B	B	B	B	B	-	-	-
22	CY427	ON989234	B	B	B	B	B	B	B	B	B	B	B	B	-	-	-
23	CY439	ON989235	A1	A1	A1	A1	A1	A1	A1	A1	A	A	A1	A1	-	-	-
24	CY443	ON989236	B	B	B	B	B	B	B	B	B	B	B	B	-	-	-
25	CY444	ON989237	A1	A1	A1	A1	A1	A1	A1	A1	A	A	A1	A1	-	-	-
26	CY447	ON989238	A1	A1	A1	A1	A1	A1	A1	A1	A	A	A1	A1	-	-	-

No.	Samples[1]	GenBank Accession Numbers	REGA 3.0[4]		COMET 2.3[5]		jpHMM[6]		SCUEAL[7]		Stanford[8]		Geno2pheno 3.4[9]		Discrepancy[10]		
			PR/RT[2]	POL[3]	PR/RT	POL	PR/RT	POL	PR/RT	POL	PR/RT	POL	PR/RT	POL	PR/RT	POL	Regions
**27**	CY448	ON989239	Rec. of B, G	Rec. of B, A1, G	Unassigned; 02_AG, 56_cpx	Unassigned; 56_cpx, 02_AG, 63_02A1, 02_AG, 71_BF1, 25_cpx, 02_AG, C, 43_02G, 02_AG, 71_BF1, 05_DF, 71_BF1, 05_DF, 02_AG, A1, B	B G	A1 B G	B, G recombinant	Complex	B + CRF02_AG	B + CRF02_AG	02_AG	B	YES	YES	YES
**28**	CY449	ON989240	A1	A1	A1	A1	A1	A1	A1	A1	A	A	A1	A1	-	-	-
**29**	CY450	ON989241	A1	A1	A1	A1	A1	A1	A1	A1	A	A	A1	A1	-	-	-
**30**	CY451	ON989242	C	C	C	C	C	C	C	C	C	C	C	C	-	-	-
**31**	CY452	ON989243	B	B	B	B	B	B	B	B	B	B	B	B	-	-	-
**32**	CY455	ON989244	A1	A1	A1	A1	A1	A1	A1	A1	A	A	01_AE	A1	YES	-	-
**33**	CY457	ON989245	B	Rec. of B, A1	B	Unassigned; B, A1	B	A1 B	B	A3, B recombinant	B	B	B	B	-	YES	YES
**34**	CY459	ON989246	F1	F1	F1	F1	F1	F1	F1	F1	F	F	F1	F1	-	-	-
**35**	CY461	ON989247	B	B	B	B	B	B	B	B	B	B	B	B	-	-	-
**36**	CY462	ON989248	CRF 06_CPX	CRF 06_CPX	06_cpx	06_cpx	A1 C G	A1 C G J	G, K recombinant	CRF06-like	CRF06_cpx	CRF06_cpx	06_CPX	06_CPX	YES	YES	YES
**37**	CY463	ON989249	01_AE	01_AE	01_AE	01_AE	01_AE	01_AE	AE	AE	CRF01_AE	CRF01_AE	01_AE	01_AE	-	-	-
**38**	CY465	ON989250	F1	F1	F1	F1	F1	F1	F1	F1	F	F	F1	F1	-	-	-
**39**	CY466	ON989251	B	B	B	B	B	B	B	B	B	B	B	B	-	-	-
**40**	CY467	OK584018	Rec. of G, A1	Rec. of G, A1, B	02_AG	Unassigned; 02_AG, 11_cpx, 01_AE, 90_BF1, B, D, B, D, B, D, B	G	A1 B G	G	Complex	CRF02_AG	CRF02_AG	02_AG	02_AG	YES	YES	YES
**41**	CY470	ON989252	02_AG	CRF 02_AG	02_AG	02_AG	G	A1 G	G	CRF02-like	CRF02_AG	CRF02_AG	02_AG	02_AG	YES	-	-
**42**	CY471	ON989253	A1	Rec. of A1, B	A1	Unassigned; A1, B	A1	A1 B	A1	A1, B recombinant	A	A	A1	A1	-	YES	YES

No.	Samples[1]	GenBank Accession Numbers	REGA 3.0[4]		COMET 2.3[5]		jpHMM[6]		SCUEAL[7]		Stanford[8]		Geno2pheno 3.4[9]		Discrepancy[10]		
			PR/RT[2]	POL[3]	PR/RT	POL	PR/RT	POL	PR/RT	POL	PR/RT	POL	PR/RT	POL	PR/RT	POL	Regions
**43**	CY472	ON989254	F1	F1	F1	F1	F1	F1	F1	F1	F	F	F1	F1	-	-	-
**44**	CY473	ON989255	B	Rec. of B, G, A1	B	Unassigned; 02_AG, 38_BF1, B, 94_cpx, 08_BC, B, 08_BC, B, 07_BC, 90_BF1, 07_BC, 52_01B, 07_BC, 90_BF1, B, 68_01B, B, 90_BF1, B	B G	A1 B G	Complex	Complex	B	B	B	B	YES	YES	YES
**45**	CY477	ON989256	B	B	B	B	B	B	B	B	B	B	B	B	-	-	-
**46**	CY478	ON989257	B	B	B	B	B	B	B	B	B	B	B	B	-	-	-
**47**	CY479	ON989258	01_AE	01_AE	01_AE	01_AE	01_AE	01_AE	AE	AE	CRF01_AE	CRF01_AE	01_AE	01_AE	-	-	-
**48**	CY480	ON989259	CRF 02_AG	CRF 02_AG	02_AG	02_AG	A1 G	A1 G	A1, G recombinant	A, G recombinant	CRF02_AG	CRF02_AG	02_AG	02_AG	-	-	-
**49**	CY482	ON989260	A2	A2	A2	A2	A2	A2	A2	A2	A2	A2	A2	A2	-	-	-
**50**	CY483	ON989261	C	C	C	C	C	C	C	C	C	C	C	C	-	-	-
**51**	CY485	ON989262	A1	A1	A1	A1	A1	A1	A1	A1	A	A	A1	A1	-	-	-
**52**	CY486	ON989263	A1	A1	A1	A1	A1	A1	A1	A1	A	A	A1	A1	-	-	-
**53**	CY487	ON989264	CRF 02_AG	CRF 02_AG	02_AG	02_AG	G	A1 G	Complex	Complex	CRF02_AG	CRF02_AG	02_AG	02_AG	YES	-	-
**54**	CY488	ON989265	Rec. of B, A1	Rec. of B, A1	Unassigned; 56_cpx, 02_AG, 90_BFI, 72_BFI, 90_BFI, 83_cpx	Unassigned; 56_cpx, 02_AG, 90_BF1, 72_BF1, 90_BF1, 83_cpx, 90_BF1, A1, 02_AG, 19_cpx, B, 90_BF1	A1 B	A1 B	Complex	Complex	B + CRF02_AG	B + CRF02_AG	02_AG	03_AB	YES	YES	YES
**55**	CY490	ON989266	A1	A1	A1	A1	A1	A1	A1	A1	A	A	01_AE	A1	YES	-	-
**56**	CY492	ON989267	A1	A1	A1	A1	A1	A1	A1	A1	A	A	01_AE	A1	YES	-	-
**57**	CY493	ON989268	A1	A1	A1	A1	A1	A1	A1	A1	A	A	01_AE	A1	YES	-	-

No.	Samples[1]	GenBank Accession Numbers	REGA 3.0[4]		COMET 2.3[5]		jpHMM[6]		SCUEAL[7]		Stanford[8]		Geno2pheno 3.4[9]		Discrepancy[10]		
			PR/RT[2]	POL[3]	PR/RT	POL	PR/RT	POL	PR/RT	POL	PR/RT	POL	PR/RT	POL	PR/RT	POL	Regions
**58**	CY494	OK283056	Rec. of G, A1	Rec. of 02_AG, G	02_AG	Unassigned; 02_AG, 11_cpx, 01_AE	G	A1 G	G	A-ancestral, G recombinant	CRF02_AG	CRF02_AG	02_AG	02_AG	YES	YES	YES
**59**	CY495	ON989269	B	B	B	B	B	B	B	B	B	B	B	B	-	-	-
**60**	CY496	ON989270	F1	F1	F1	F1	F1	F1	F1	F1	F	F	F1	F1	-	-	-
**61**	CY497	ON989271	G	Rec. of A1, G	02_AG	02_AG	G	A1 G	G	Complex	CRF02_AG	CRF02_AG	02_AG	02_AG	YES	-	-
**62**	CY498	ON989272	B	B	B	B	B	B	B	B	B	B	B	B	-	-	-
**63**	CY500	ON989273	A1	A1	A1	A1	A1	A1	A1	A1	A	A	A1	A1	-	-	-
**64**	CY504	ON989274	F1	F1	F1	F1	F1	F1	F1	F1	F	F	F1	F1	-	-	-
**65**	CY508	ON989275	G	Rec. of G, K	G	Unassigned; G, A1, D	G	B G	G	G, J recombinant	G	G	G	G	-	YES	YES
**66**	CY509	ON989276	B	B	B	B	B	B	B	B	B	B	B	B	-	-	-
**67**	CY510	ON989277	B	B	B	B	B	B	B	B	B	B	B	B	-	-	-
**68**	CY515	ON989278	Rec. of F1, B	Rec. of B, F1	F1	Unassigned; B, F1, A1, F1, F2	B F1	B F1	Complex	B, F1 recombinant	B	B	12_BF	B	YES	YES	YES
**69**	CY516	ON989279	CRF 03_AB	CRF 03_AB	03_AB	03_AB	A1 B	A1 B	CRF03	CRF03	CRF03_AB	A + B	03_AB	03_AB	-	-	-
**70**	CY519	ON989280	F1	Rec. of F1, A1	Unassigned; 71_BF1, A1	Unassigned; F1, A1, 01_AE, A1, G	F1	A1 F1	F1	Complex	F	F	F1	F2	YES	YES	YES
**71**	CY520	OK283057	Rec. of G, A1	Rec. of 02_AG, G	02_AG	Unassigned; 02_AG, 11_cpx, 01_AE	G	A1 G	G	Complex	CRF02_AG	CRF02_AG	02_AG	02_AG	YES	YES	YES
**72**	CY523	ON989281	A1	A1	A1	A1	A1	A1	A1	A1	A	A	A1	A1	-	-	-
**73**	CY525	ON989282	Rec. of G, B	Rec. of B, A1, G	Unassigned; 02_AG, 56_cpx	Unassigned; 02_AG, 56_cpx, 90_BF1, B, 90_BF1, B, 56_cpx, B, 56_cpx, B, 39_BF, 51_01B, B, 51_01B, B, 90_BF1, 56_cpx,	B C G	A1 B G	Complex	Complex	B + CRF02_AG	B + CRF02_AG	02_AG	B	YES	YES	YES

No.	Samples[1]	GenBank Accession Numbers	REGA 3.0[4]		COMET 2.3[5]		jpHMM[6]		SCUEAL[7]		Stanford[8]		Geno2pheno 3.4[9]		Discrepancy[10]		
			PR/RT[2]	POL[3]	PR/RT	POL	PR/RT	POL	PR/RT	POL	PR/RT	POL	PR/RT	POL	PR/RT	POL	Regions
						90_BF1											
**74**	CY526	ON989283	Rec. of G, B	Rec. of B, A1, G	Unassigned; 02_AG, 56_cpx	Unassigned; 02_AG, 56_cpx, 90_BF1, B, 90_BF1, B, 56_cpx, B, 39_BF, 90_BF1, 39_BF, B, 69_01B, 90_BF1, 56_cpx	B G	A1 B G	Complex	Complex	B + CRF02_AG	B + CRF02_AG	02_AG	B	YES	YES	YES
**75**	CY528	ON989284	B	B-like	B	B	B	B	B	B	B	B	B	B	-	-	-
**76**	CY529	ON989285	Rec. of G, B	Rec. of B, A1, G	Unassigned; 02_AG, 56_cpx	Unassigned; 02_AG, 56_cpx, 90_BF1, B, 90_BF1, B, 56_cpx, B, 56_cpx, B, 39_BF, 90_BF1, B, 90_BF1, 56_cpx	B C G	A1 B G	Complex	Complex	B + CRF02_AG	B + CRF02_AG	02_AG	B	YES	YES	YES
**77**	CY530	ON989286	CRF 02_AG	CRF 02_AG	02_AG	02_AG	A1 G	A1 G	Complex	Complex	CRF02_AG	CRF02_AG	02_AG	02_AG	-	-	-
**78**	CY533	OK283058	Rec. of G, A1	Rec. of 02_AG, G, A1	02_AG	Unassigned; 02_AG, 11_cpx, 01_AE	G	A1 G	G	A, G recombinant	CRF02_AG	CRF02_AG	02_AG	02_AG	YES	YES	YES
**79**	CY534	ON989287	A1	A1	A1	A1	A1	A1	A1	A1	A	A	A1	A1	-	-	-
**80**	CY535	ON989288	CRF 02_AG	CRF 02_AG	02_AG	02_AG	A1 D G	A1 C G	Complex	Complex	CRF02_AG	CRF02_AG	02_AG	02_AG	YES	YES	YES
**81**	CY537	ON989289	Rec. of G, B	Rec. of B, A1, G	Unassigned; 02_AG, 90_BF1, 31_BC, 56_cpx	Unassigned; 02_AG, 90_BF1, 31_BC, 56_cpx, 90_BF1, B, 90_BF1, B,	B C G	A1 B G	Complex	Complex	B + CRF02_AG	B + CRF02_AG	02_AG	B	YES	YES	YES

No.	Samples[1]	GenBank Accession Numbers	REGA 3.0[4]		COMET 2.3[5]		jpHMM[6]		SCUEAL[7]		Stanford[8]		Geno2pheno 3.4[9]		Discrepancy[10]		
			PR/RT[2]	POL[3]	PR/RT	POL	PR/RT	POL	PR/RT	POL	PR/RT	POL	PR/RT	POL	PR/RT	POL	Regions
						56_cpx, B, 39_BF, 90_BF1, 39_BF, B, 69_01B, 90_BF1, 56_cpx											
**82**	CY538	ON989290	02_AG	Rec. of 02_AG, A1	02_AG	02_AG	G	A1 G	G	Complex	CRF02_AG	CRF02_AG	02_AG	02_AG	YES	YES	YES
**83**	CY539	ON989291	A1	A1	A1	A1	A1	A1	A1	A1	A	A	A1	A1	-	-	-
**84**	CY541	ON989292	CRF 04_CPX	CRF 04_CPX	F1 (check for 04_cpx)	04_cpx	A1 C	A1 C	Complex	Complex	CRF04_cpx	CRF04_cpx	04_CPX	04_CPX	YES	YES	YES
**85**	CY542	ON989293	01_AE	01_AE	01_AE	01_AE	01_AE	01_AE	AE, B recombinant	AE, D recombinant	CRF01_AE	CRF01_AE	01_AE	01_AE	YES	YES	YES
**86**	CY543	ON989294	A1	A1	A1	A1	A1	A1	A1	A1	A	A	01_AE	01_AE	YES	YES	YES
**87**	CY544	ON989295	A1	A1	A1	A1	A1	A1	A1	A1	A	A	A1	A1	-	-	-
**88**	CY545	ON989296	A1	A1	A1	A1	A1	A1	A1	A1	A	A	A1	A1	-	-	-
**89**	CY546	ON989297	A1	A1	A1	A1	A1	A1	A1	A1	A	A	A1	A1	-	-	-
**90**	CY550	ON989298	B	B	B	B	B	B	B	B	B	B	B	B	-	-	-
**91**	CY551	ON989299	B	B	B	B	B	B	B, G recombinant	B	B	B	B	B	YES	-	-
**92**	CY552	ON989300	CRF 02_AG	CRF 02_AG	A1 (check for 63_02A1)	A1 (check for 02_AG)	A1	A1 G	A, G recombinant	CRF02-like	A + CRF02_AG	CRF02_AG	02_AG	02_AG	YES	-	-
**93**	CY553	ON989301	F1	F1	F1	F1	F1	F1	F1	F1	F	F	F1	F1	-	-	-
**94**	CY554	ON989302	CRF 02_AG	CRF 02_AG	02_AG	02_AG	A1 G	A1 G	Complex	Complex	CRF02_AG	CRF02_AG	02_AG	02_AG	-	-	-
**95**	CY555	ON989303	A1	A1	A1	A1	A1	A1	A1	A1	A	A	01_AE	01_AE	YES	YES	YES
**96**	CY556	ON989304	CRF 02_AG	CRF 02_AG	02_AG	02_AG	G	A1 G	G	Complex	CRF02_AG	CRF02_AG	02_AG	02_AG	YES	-	-
**97**	CY557	ON989305	A1	A1	A1	A1	A1	A1	A1	A1	A	A	01_AE	A1	YES	-	-
**98**	CY559	ON989306	CRF 02_AG	CRF 02_AG	02_AG	02_AG	G	A1 G	AE, G recombinant	Complex	CRF02_AG	CRF02_AG	02_AG	02_AG	YES	-	-
**99**	CY560	ON989307	B	B	B	B	B	B	B	B	B	B	B	B	-	-	-
**100**	CY562	ON989308	B	B	B	B	B	B	B	B	B	B	B	B	-	-	-
**101**	CY563	ON989309	B	B	B	B	B	B	B	B	B	B	B	B	-	-	-
**102**	CY564	ON989310	A1	A1	A1	A1	A1	A1	A1	A1	A	A	A1	A1	-	-	-
**103**	CY565	ON989311	F1	F1	F1	F1	F1	F1	F1	F1	F	F	F1	F1	-	-	-
**104**	CY571	ON989312	CRF 02_AG	CRF 02_AG	02_AG	02_AG	A1 G	A1 G	A2, G recombinant	Complex	CRF02_AG	CRF02_AG	02_AG	02_AG	YES	-	-
**105**	CY572	ON989313	B	B	B	B	B	B	B	B	B	B	B	B	-	-	-

No.	Samples[1]	GenBank Accession Numbers	REGA 3.0[4]		COMET 2.3[5]		jpHMM[6]		SCUEAL[7]		Stanford[8]		Geno2pheno 3.4[9]		Discrepancy[10]		
			PR/RT[2]	POL[3]	PR/RT	POL	PR/RT	POL	PR/RT	POL	PR/RT	POL	PR/RT	POL	PR/RT	POL	Regions
**106**	CY575	ON989314	A1	A1	A1	A1	A1	A1	A1	A1	A	A	A1	A1	-	-	-
**107**	CY576	ON989315	CRF 02_AG	CRF 02_AG	02_AG	02_AG	A1 G	A1 B G	Complex	Complex	CRF02_AG	CRF02_AG	02_AG	02_AG	-	YES	YES
**108**	CY579	ON989316	CRF 02_AG	CRF 02_AG	02_AG	02_AG	C G	A1 G	Complex	CRF02-like	CRF02_AG	CRF02_AG	02_AG	02_AG	YES	-	-
**109**	CY580	ON989317	D	D-like	D	D	D	D	D	D	D	D	D	D	-	-	-
**110**	CY581	ON989318	CRF 02_AG	Rec. of 02_AG, G, A1	02_AG	02_AG	A1 G	A1 G	G	A, G recombinant	CRF02_AG	CRF02_AG	02_AG	02_AG	YES	YES	YES
**111**	CY583	ON989319	B	B	B	B	B	B	B	B	B	B	B	B	-	-	-
**112**	CY584	ON989320	B	Rec. of B, A1	B	Unassigned; B, A1	B	A1 B	B, D recombinant	A1, B recombinant	B	B	B	B	YES	YES	YES
**113**	CY585	ON989321	02_AG	CRF 02_AG	02_AG	02_AG	A1 G	A1 G	A1, G recombinant	Complex	CRF02_AG	CRF02_AG	02_AG	02_AG	-	-	-
**114**	CY587	ON989322	A1	A1	A1	A1	A1	A1	A1	A1	A	A	A1	A1	-	-	-
**115**	CY591	ON989323	A1	A1	A1	A1	A1	A1	A1	A1	A	A	A1	A1	-	-	-
**116**	CY593	ON989324	B	B	B	B	B	B	B	B	B	B	B	B	-	-	-
**117**	CY594	ON989325	B	B	B	B	B	B	B	B	B	B	B	B	-	-	-
**118**	CY599	ON989326	CRF 02_AG	CRF 02_AG	02_AG	02_AG	G	A1 G	Complex	Complex	CRF02_AG	CRF02_AG	02_AG	02_AG	YES	-	-
**119**	CY601	ON989327	C	C	C	C	C	C	C	C	C	C	C	C	-	-	-
**120**	CY602	ON989328	CRF 02_AG	CRF 02_AG	02_AG	A1 (check for 02_AG)	G	A1 G	Complex	Complex	CRF02_AG	CRF02_AG	02_AG	02_AG	YES	-	-
**121**	CY604	ON989329	F1	F1	F1	F1	F1	F1	F1	F1	F	F	F1	F1	-	-	-
**122**	CY605	ON989330	A1	A1	A1	A1	A1	A1	A1	A1	A	A	A1	A1	-	-	-
**123**	CY607	ON989331	B	B	B	B	B	B	B	B	B	B	B	B	-	-	-
**124**	CY609	ON989332	B	B	B	B	B	B	B	B	B	B	B	B	-	-	-
**125**	CY611	ON989333	Rec. of 18_cpx, G	CRF 18_cpx	F1 (check for 18_cpx)	18_cpx	B G	A1 C F1 G	AE, G recombinant	G, K recombinant	CRF18_cpx	CRF18_cpx	K	02_AG	YES	YES	YES
**126**	CY612	ON989334	CRF 06_CPX	CRF 06_CPX	06_cpx	06_cpx	C G	A1 C G J	CRF06-like	Complex	CRF06_cpx	CRF06_cpx	06_CPX	06_CPX	YES	YES	YES
**127**	CY614	OK283059	Rec. of G, A1	Rec. of 02_AG, G, A1	02_AG	Unassigned; 02_AG, 11_cpx, 01_AE	G	A1 G	G	Complex	CRF02_AG	CRF02_AG	02_AG	02_AG	YES	YES	YES
**128**	CY615	ON989335	A1	A1	A1	A1	A1	A1	A1	A1	A	A	01_AE	01_AE	YES	YES	YES
**129**	CY616	ON989336	B	B	B	B	B	B	B	B	B	B	B	B	-	-	-
**130**	CY617	ON989337	F1	F1	F1	F1	F1	F1	F1	F1	F	F	F1	F1	-	-	-
**131**	CY619	ON989338	G	G	G	G	G	G	G	G	G	G	G	G	-	-	-
**132**	CY620	ON989339	B	Rec. of B, A1	B	Unassigned; B, A1	B	A1 B	B, D recombinant	Complex	B	B	B	B	YES	YES	YES

No.	Samples[1]	GenBank Accession Numbers	REGA 3.0[4]		COMET 2.3[5]		jpHMM[6]		SCUEAL[7]		Stanford[8]		Geno2pheno 3.4[9]		Discrepancy[10]		
			PR/RT[2]	POL[3]	PR/RT	POL	PR/RT	POL	PR/RT	POL	PR/RT	POL	PR/RT	POL	PR/RT	POL	Regions
**133**	CY624	ON989340	CRF 02_AG	CRF 02_AG	02_AG	02_AG	C G	A1 G	Complex	CRF02-like	CRF02_AG	CRF02_AG	02_AG	02_AG	YES	-	-
**134**	CY625	ON989341	Rec. of B, G	Rec. of B, A1, G	Unassigned; 02_AG, B, 56_cpx	Unassigned; 02_AG, B, 56_cpx, 90_BF1, B, 56_cpx, B, 51_01B, B, 69_01B, 90_BF1	A1 B G	A1 B G	Complex	Complex	B + CRF02_AG	B + CRF02_AG	02_AG	B	YES	YES	YES

The cells highlighted in light-pink color show the discrepant subtyping of *'PR/RT'* regions (corresponding to nucleotides 2253 to 3359 on the HXB2 genome), and the cells highlighted in light-gray color show the discrepant subtyping of *'POL'* regions (corresponding to nucleotides 2253 to 5250 on HXB2 genome). [1] 'Samples' corresponds to the coded naming of study subject samples that contains five characters; e.g., in CY001, the first two characters, CY, denote Cyprus, the country of origin. The next three characters, 001, denote the study subject's number. [2] *'PR/RT'* denotes nucleotide sequences of *protease* and partial *reverse transcriptase* genes (corresponding to nucleotides 2253 to 3359 on the HXB2 genome). [3] *'POL'* denotes nucleotide sequences of *protease, reverse transcriptase, integrase,* and partial *vif* genes (corresponding to nucleotides 2253 to 5250 on the HXB2 genome). [4] REGA HIV-1 subtyping tool version 3.0 identifies the subtypes via using phylogenetic methods; the various recombinations are analyzed with bootscanning methods. It is available at: http://dbpartners.stanford.edu:8080/RegaSubtyping/stanford-hiv/typingtool/, accessed on 14 October 2022. [5] COMET 2.3 is the abbreviation for "COntext-based Modeling for Expeditious Typing", while version 2.3 has additional support for HIV-1 nucleotide sequences for CRFs as well as URFs. It is available at: https://comet.lih.lu/, accessed on 14 October 2022. [6] jpHMM ('jumping profile hidden Markov model') predicts the genomic recombination of HIV-1 and the accurate breakpoints between the subtypes. It is available online at: http://jphmm.gobics.de/, accessed on 14 October 2022. [7] SCUEAL is an algorithm that can subtype only *pol* sequences. It is available at: http://classic.datamonkey.org/dataupload_scueal.php, accessed on 14 October 2022. [8] Stanford University curates the HIV Drug Resistance Database, which provides the public with the HIVdb Program. Its main function is to accept *pol* sequences and, in return, predict the resistance to various drugs, subtyping the sequence in parallel. It is available at: https://hivdb.stanford.edu/hivdb/by-sequences/, accessed on 14 October 2022. [9] Geno2pheno (resistance) 3.4 aligns HIV-1 *pol* gene nucleotide sequences to the HXB2 genome in order to predict resistance to ART drugs. It is available at: https://www.geno2pheno.org/index.php, accessed on 14 October 2022. [10] 'Discrepancy' denotes the disagreements in subtyping generated by the aforementioned subtyping tools. Discrepancy in the *'PR/RT'* region indicates the disagreements in the subtyping of each *PR/RT* nucleotide sequence (corresponding to nucleotides 2253 to 3359 on the HXB2 genome) among different subtyping tools. Discrepancy in the *'POL'* region indicates the disagreements in the subtyping of each *pol* nucleotide sequence (corresponding to nucleotides 2253 to 5250 on the HXB2 genome) among different subtyping tools. Discrepancy in 'Regions' denotes the verdict of discrepant result, where the disagreements in both *'PR/RT'* and *'POL'* regions or *'POL'* region only indicate that there are discrepancies among the subtyping tools. Samples identified as discrepant in 'Regions' were selected to proceed with near-full-length HIV-1 genome sequencing.
